# Supplementary material for: Berberine protects hepatocyte from hypoxia/reoxygenation-induced injury through inhibiting circDNTTIP2
Source: PeerJ. 2023 Sep 25;11:e16080. doi: 10.7717/peerj.16080 (PMC10538280; doi:10.7717/peerj.16080)
Supplement: Supplemental Information 1 [file peerj-11-16080-s001.pdf]

Supplementary Table1. primers

Primer name	qPCR primer sequence (5’→3’)
hsa_circ_0005199-Forward	AGTCTGACAGCCACATCACA
hsa_circ_0005199-Reverse	TGACTCCTTCACTGCTCTGG
hsa_circ_0110306-Forward	TGAGGCTTCAGAAATTGTCCAG
hsa_circ_0110306-Reverse	CCAGCTTCCCATCTTTGTTGA
hsa_circ_0000117-Forward	TAGGACATATGGGTGGGGAC
hsa_circ_0000117-Reverse	GCCTTCTCATGATCAGCTCG
hsa_circ_0008339-Forward	CTGTCTATCACCAGCCAGCA
hsa_circ_0008339-Reverse	TGGCAGCTTTTACCACTCCA
hsa_circ_0111350-Forward	ACAACAGTCAGACTCAGCCA
hsa_circ_0111350-Reverse	AGCATCACCTCCTTTTCCCA
hsa_circ_0016601-Forward	GCAGTTACAGCAAGCTACCC
hsa_circ_0016601-Reverse	TCTTGGTTTCCTCCTTGTCCA
hsa_circ_0013218-Forward	TGGAAGAGGAAGACAAGGCA
hsa_circ_0013218-Reverse	TGCGCTTGAATCCCATTAGC
hsa_circ_0112397-Forward	GCTGCTCTTTCGAATCGTCA
hsa_circ_0112397-Reverse	ACATCTGGCTGGTCATGAGT
hsa_circ_0012152-Forward	ATCGGGAAGCCTCATCTAGC
hsa_circ_0012152-Reverse	CAGAGGGTTGGGAAGGTAGG
hsa_circ_0002563-Forward	AATCAACCCTGCTACCGGAA
hsa_circ_0002563-Reverse	TGTCTTGCCACTTCCTGTCT
DNTTIP2-Forward	GGAAGAGGGAAGTCGTGGTG
DNTTIP2-Reverse	GTCCCCTTTGGTAGTGAGCC
IL-1β-Forward	TGAGCTCGCCAGTGAAATGA
IL-1β-Reverse	AACACGCAGGACAGGTACAG
IL-6-Forward	GTCCAGTTGCCTTCTCCCTGG
IL-6-Reverse	CCCATGCTACATTTGCCGAAG
IL-10-Forward	TTGCAAAAGAAGGCATGCACAG
IL-10-Reverse	ATAGAGTCGCCACCCTGATG
TNFα-Forward	CACAGTGAAGTGCTGGCAAC
TNFα-Reverse	ACATTGGGTCCCCCAGGATA
Caspase 3-Forward	GCTCATACCTGTGGCTGTGT
Caspase 3-Reverse	TCTGTTGCCACCTTTCGGTT
β-actin-Forward	GGGAAATCGTGCGTGACATTAAG
β-actin-Reverse	TGTGTTGGCGTACAGGTCTTTG
