# Supplementary figures and images for: Berberine protects hepatocyte from hypoxia/reoxygenation-induced injury through inhibiting circDNTTIP2
Source: PeerJ. 2023 Sep 25;11:e16080. doi: 10.7717/peerj.16080 (PMC10538280; doi:10.7717/peerj.16080)

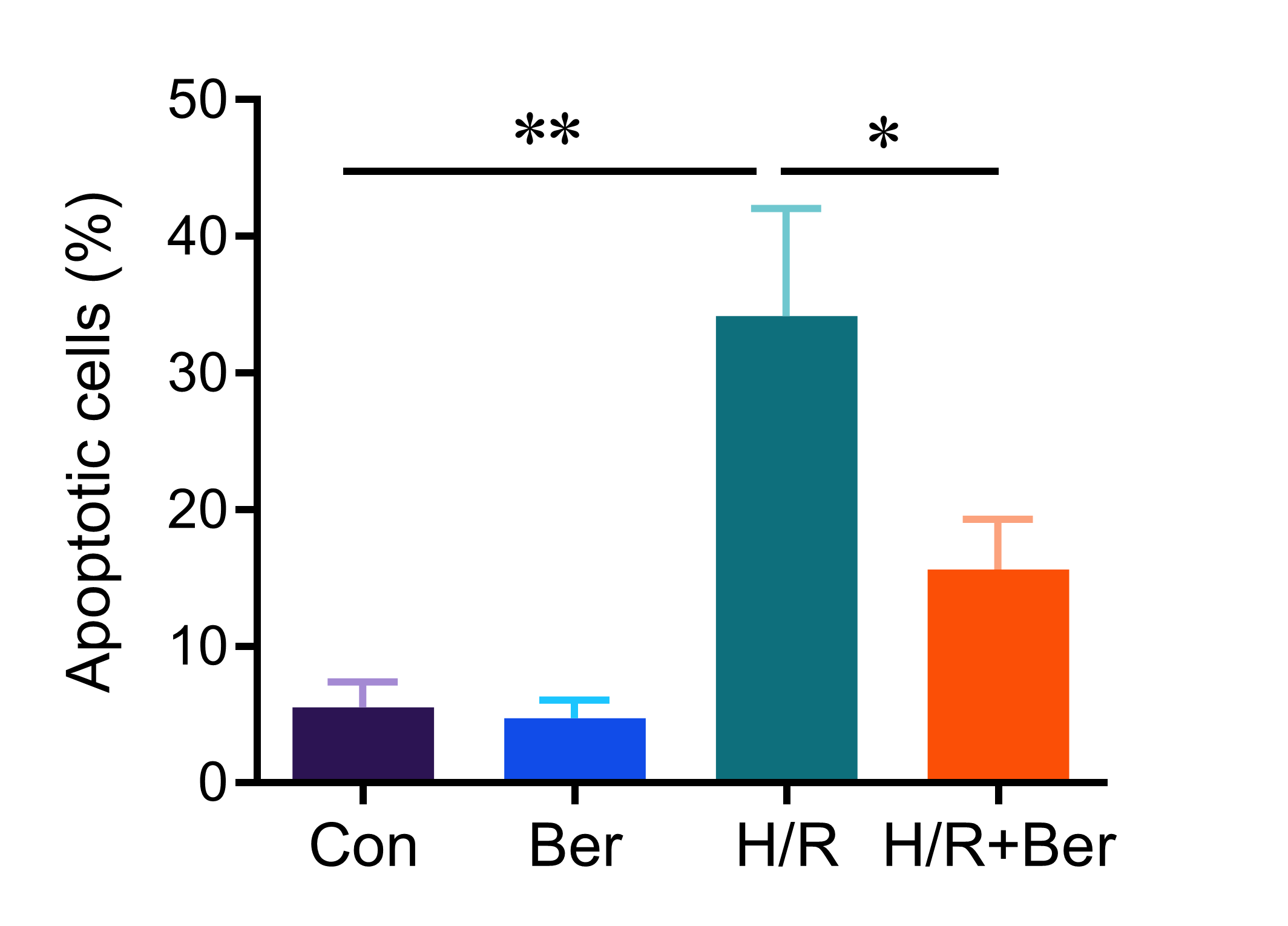

Supplement: Supplemental Information 3 [file peerj-11-16080-s003.zip › Figure1/apotosis 1.tif]

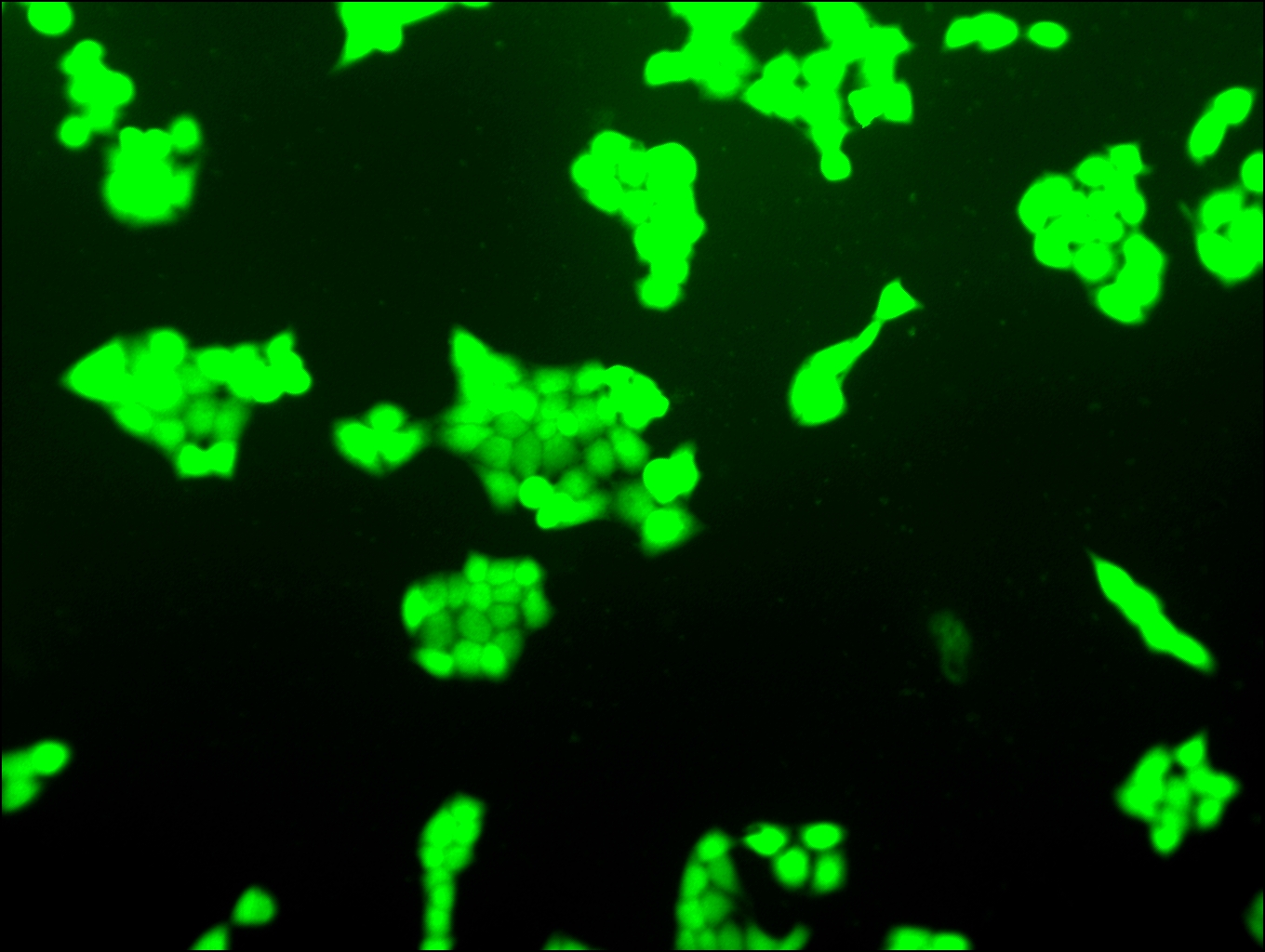

Supplement: Supplemental Information 3 [file peerj-11-16080-s003.zip › Figure1/ROS HR.jpg]

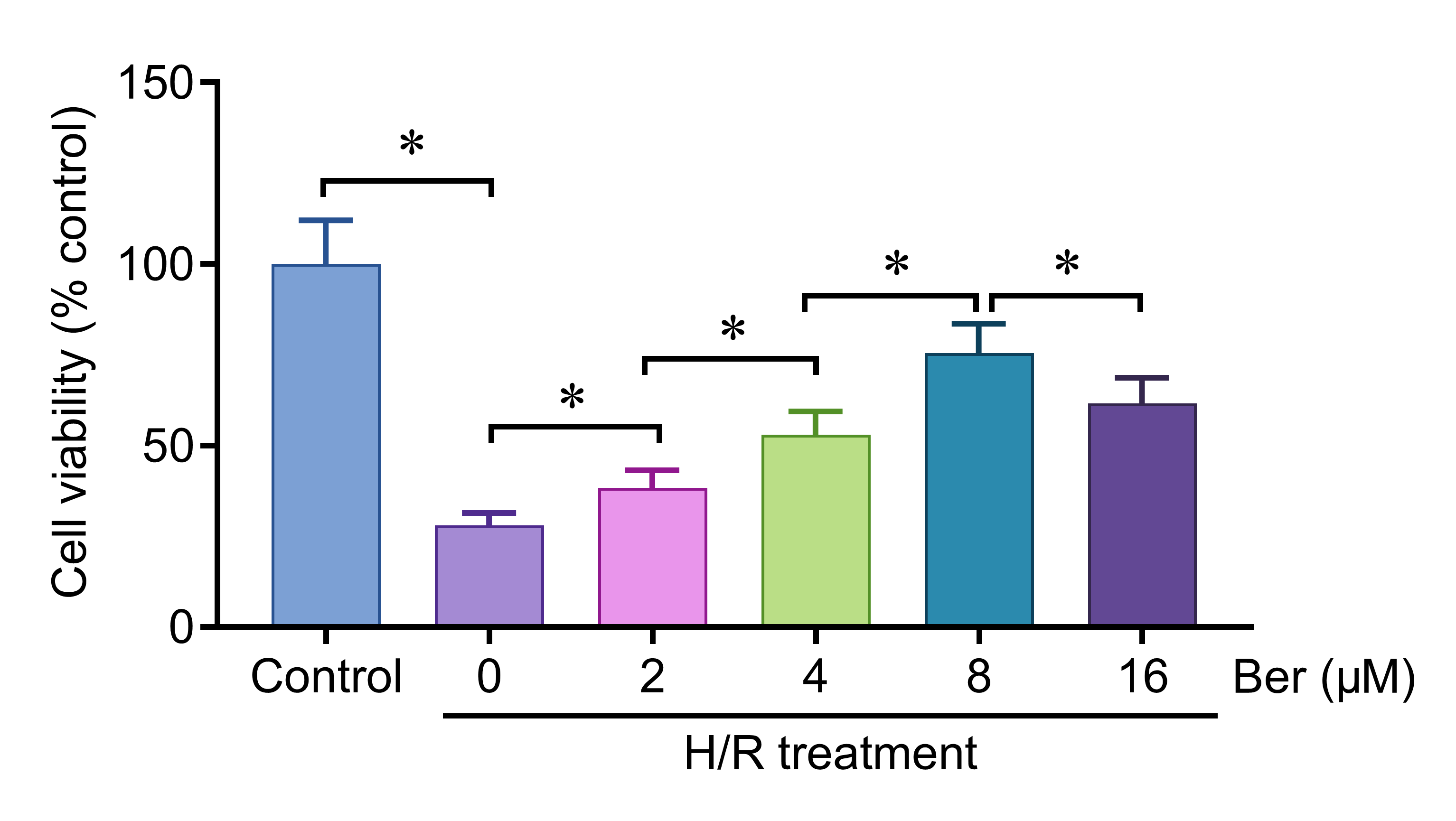

Supplement: Supplemental Information 3 [file peerj-11-16080-s003.zip › Figure1/cell viability.tif]

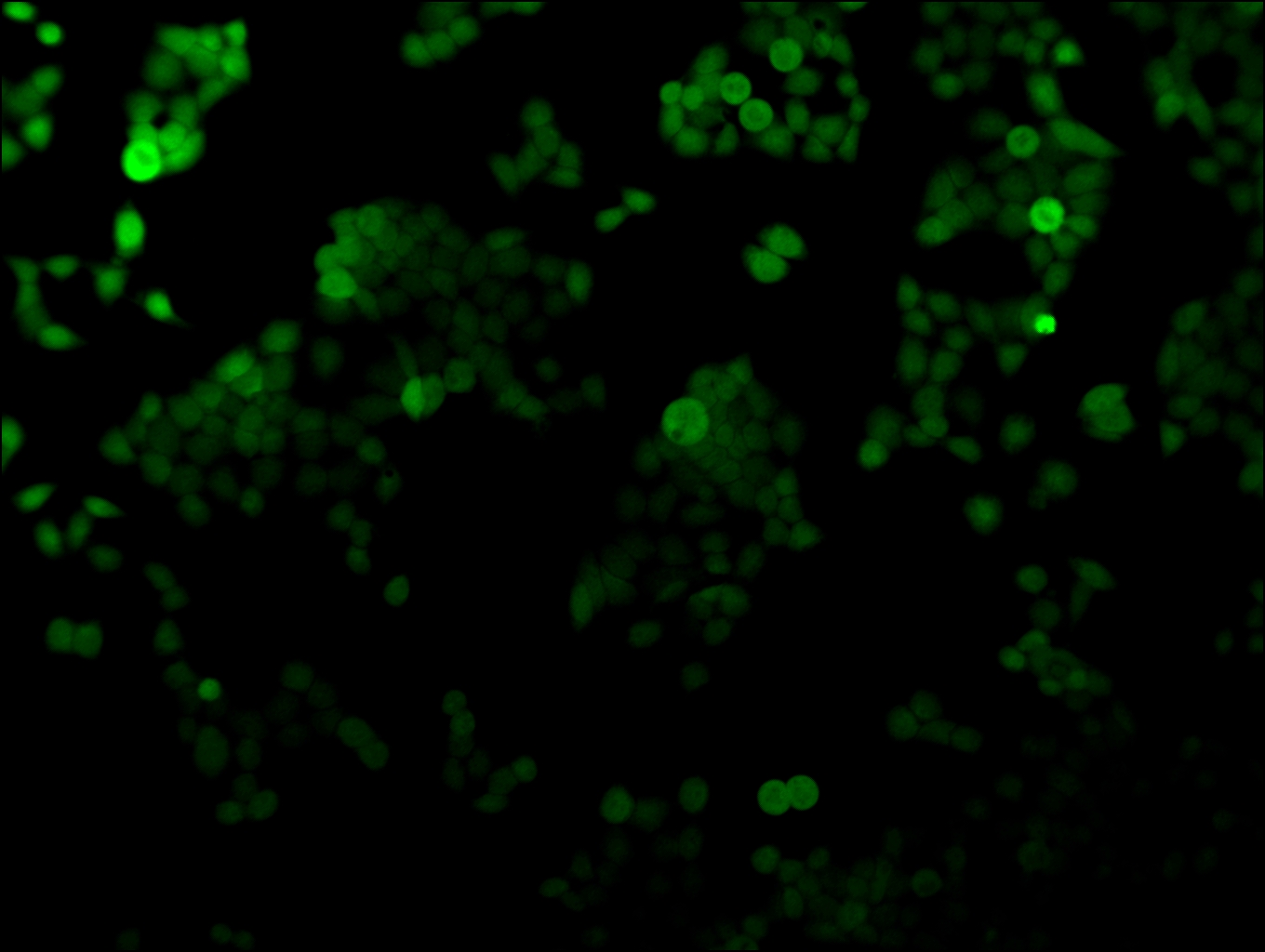

Supplement: Supplemental Information 3 [file peerj-11-16080-s003.zip › Figure1/ROS ber.jpg]

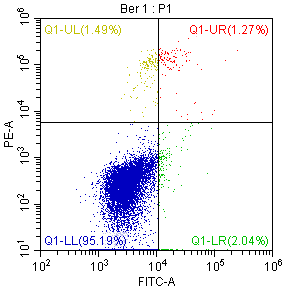

Supplement: Supplemental Information 3 [file peerj-11-16080-s003.zip › Figure1/ap ber.tif]

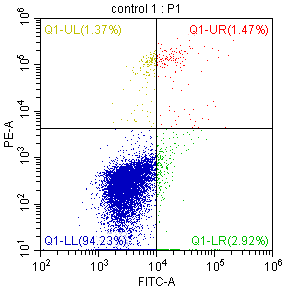

Supplement: Supplemental Information 3 [file peerj-11-16080-s003.zip › Figure1/ap control.tif]

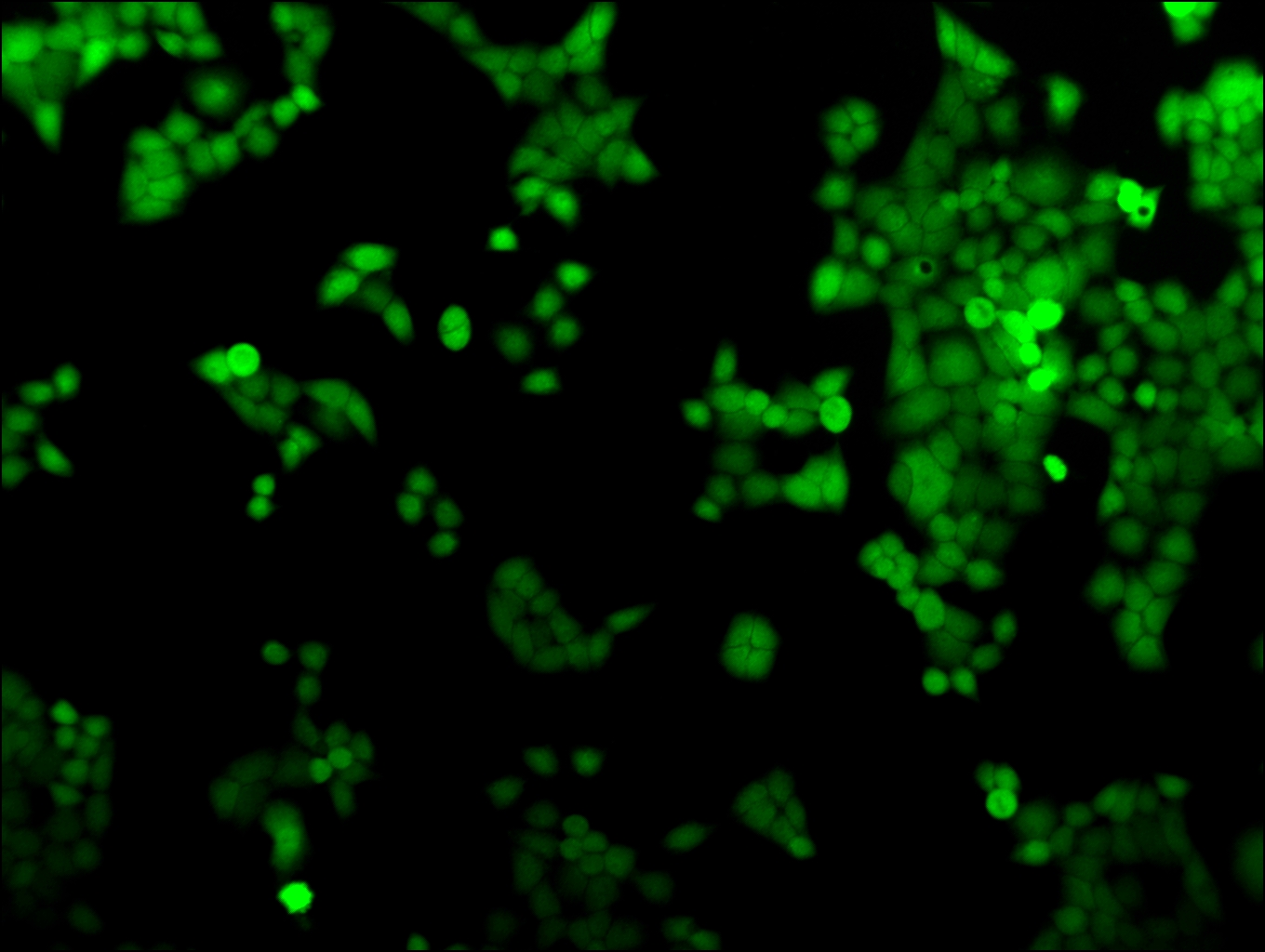

Supplement: Supplemental Information 3 [file peerj-11-16080-s003.zip › Figure1/ROS control.jpg]

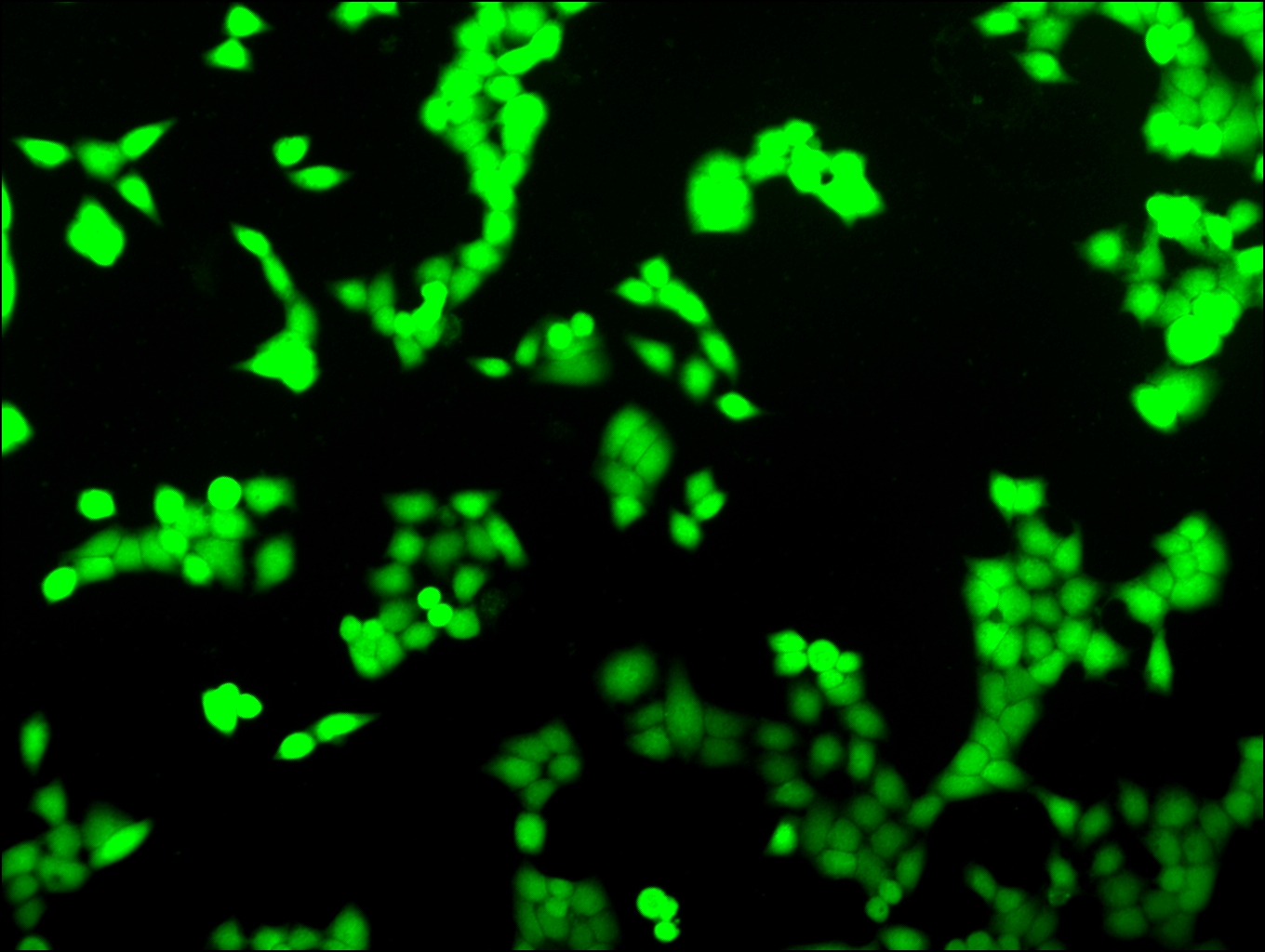

Supplement: Supplemental Information 3 [file peerj-11-16080-s003.zip › Figure1/ROS HR ber.jpg]

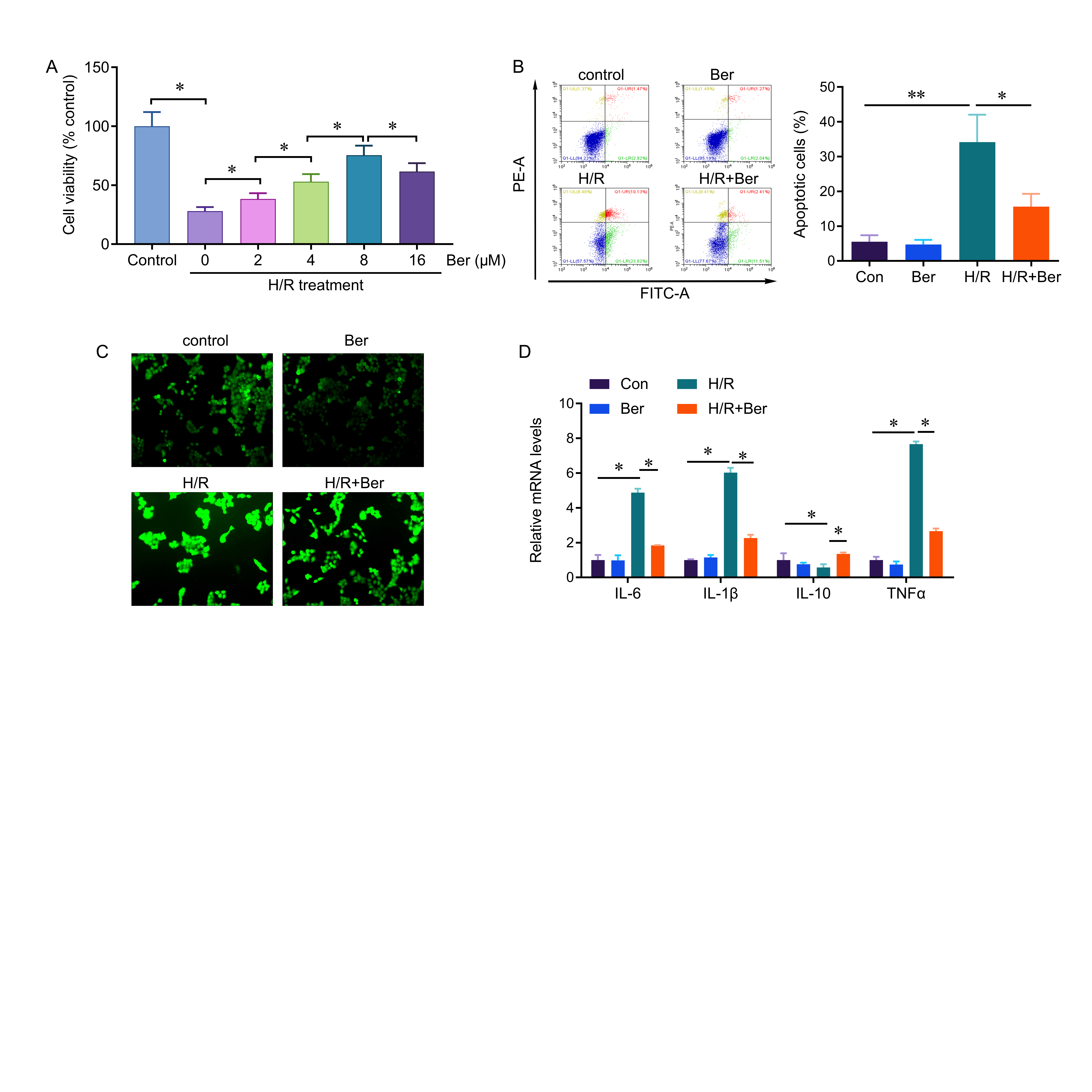

Supplement: Supplemental Information 3 [file peerj-11-16080-s003.zip › Figure1/Figure1-1.tif]

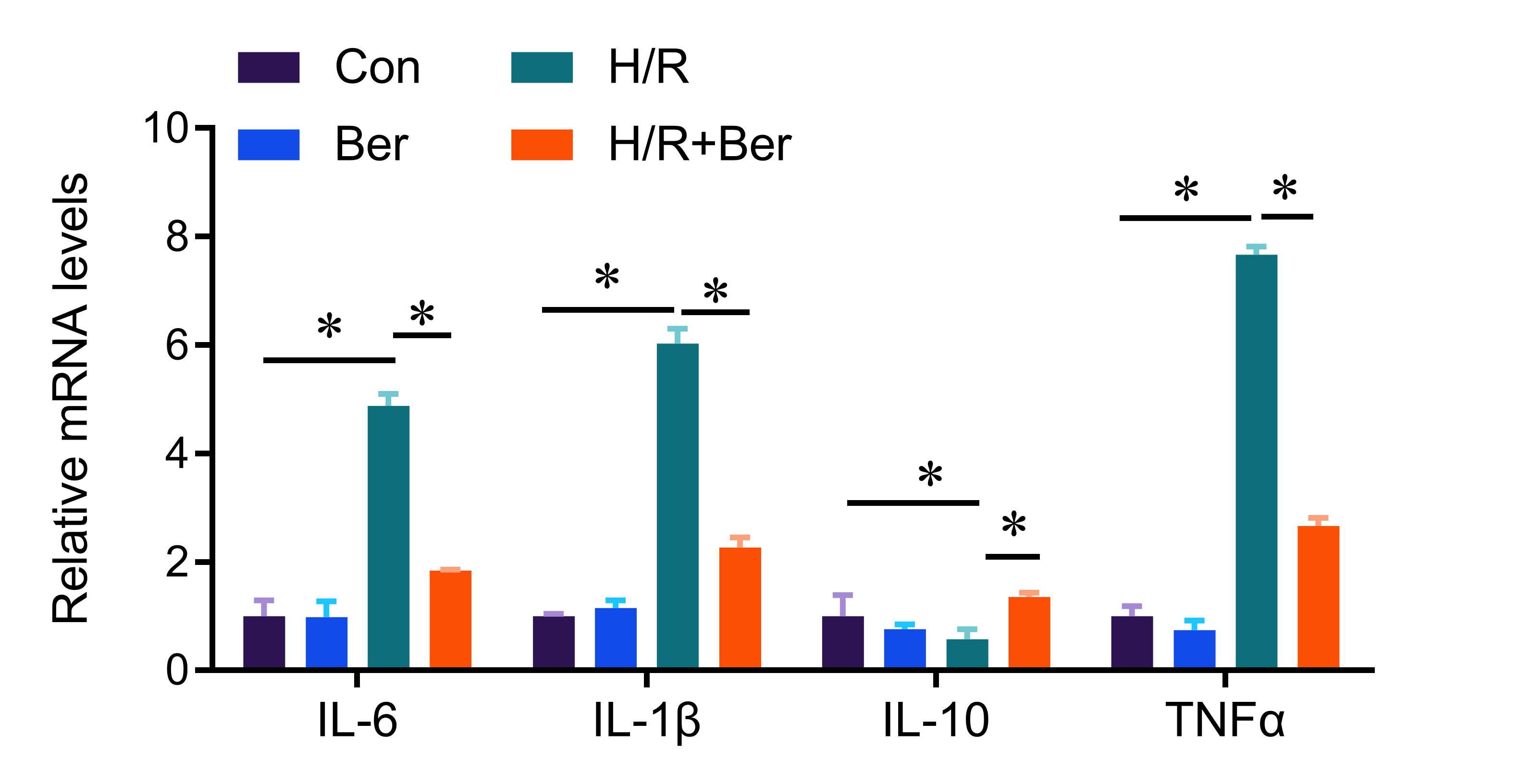

Supplement: Supplemental Information 3 [file peerj-11-16080-s003.zip › Figure1/mrna Figure1.tif]

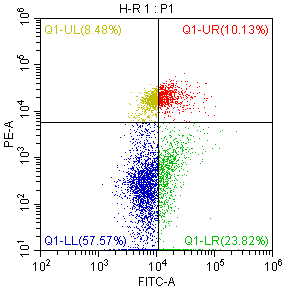

Supplement: Supplemental Information 3 [file peerj-11-16080-s003.zip › Figure1/ap HR.tif]

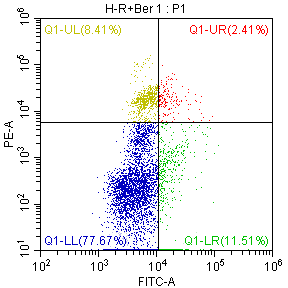

Supplement: Supplemental Information 3 [file peerj-11-16080-s003.zip › Figure1/ap HR+ber.tif]

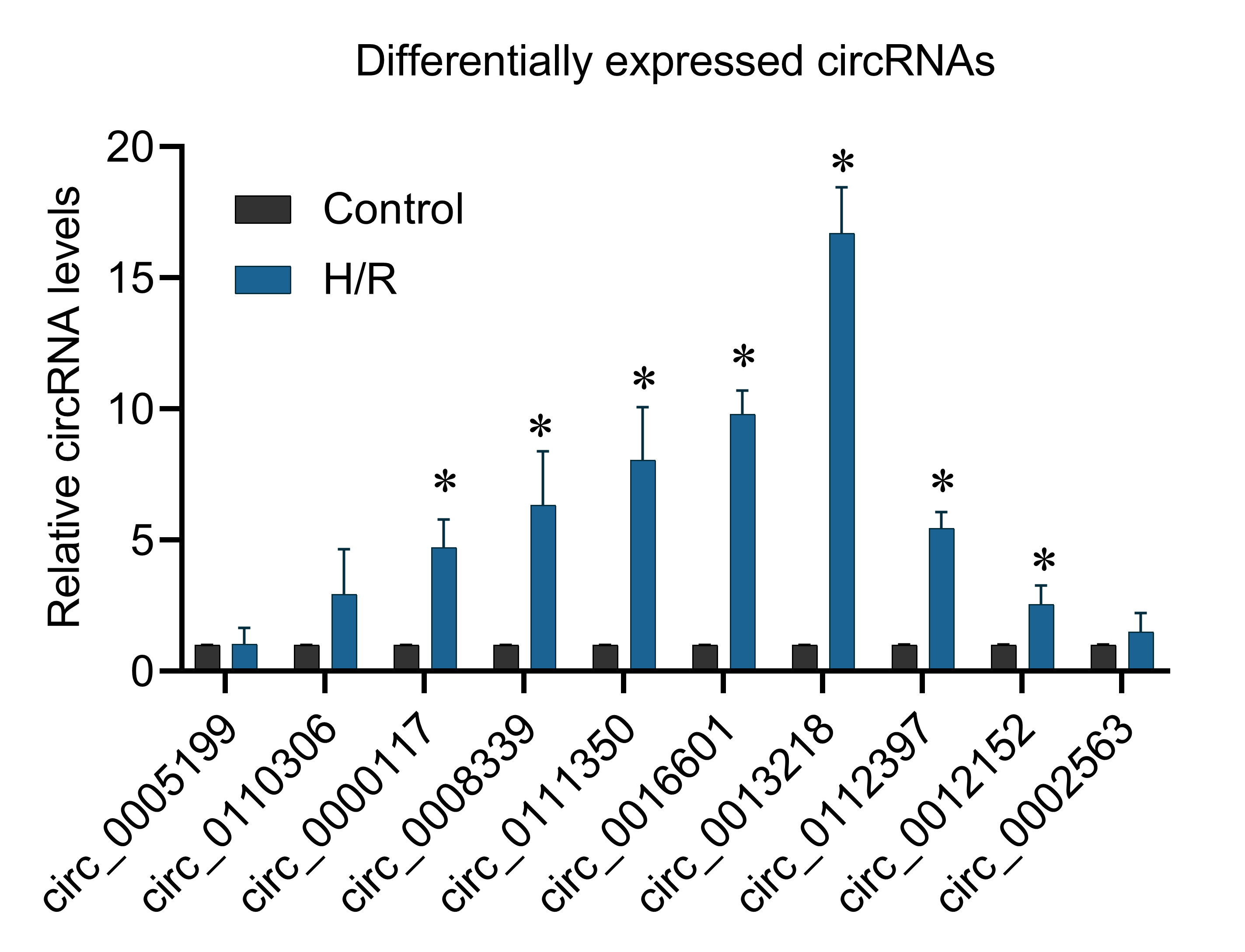

Supplement: Supplemental Information 4 [file peerj-11-16080-s004.zip › Figure2/differentially expressed circrnas.tif]

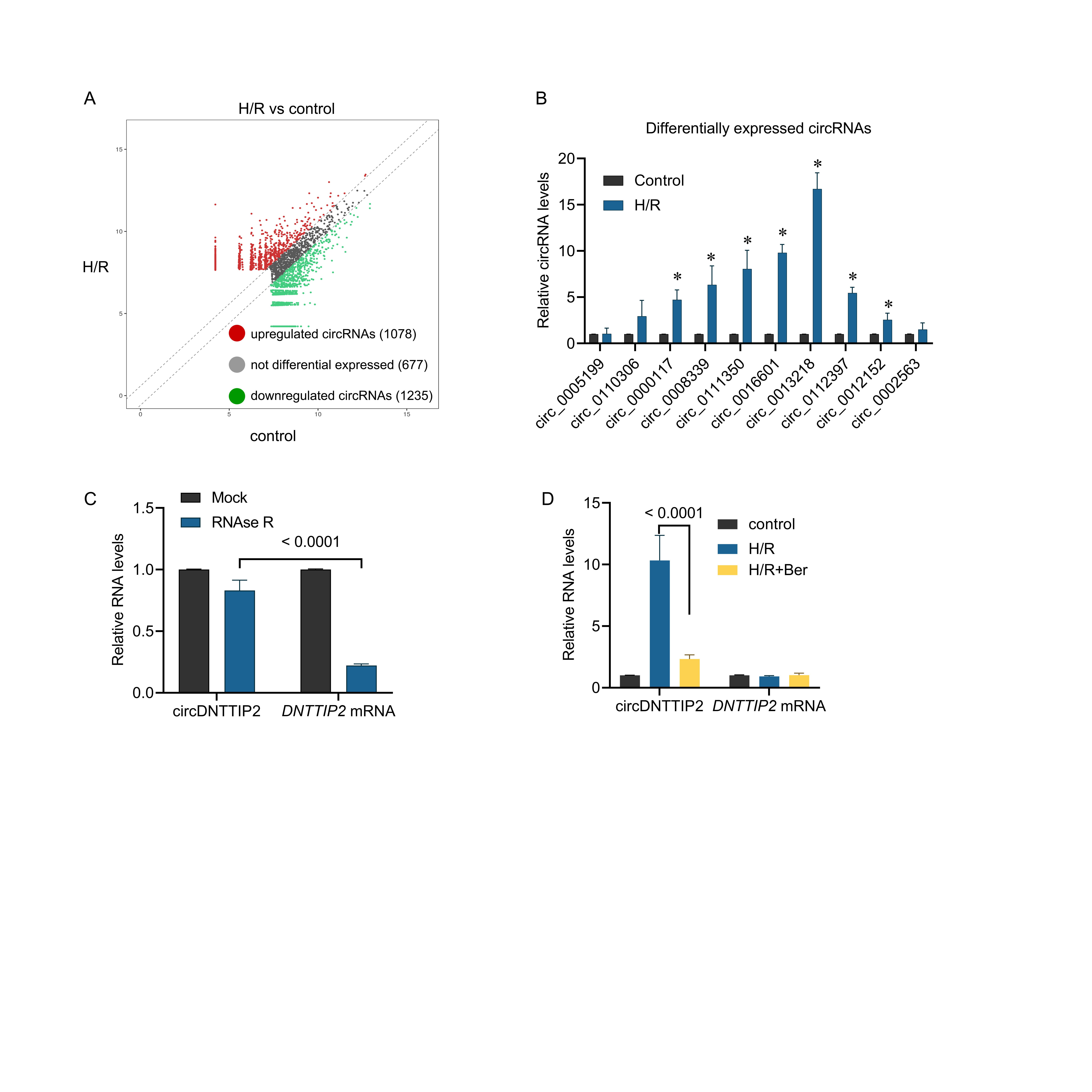

Supplement: Supplemental Information 4 [file peerj-11-16080-s004.zip › Figure2/Figure2-1.tif]

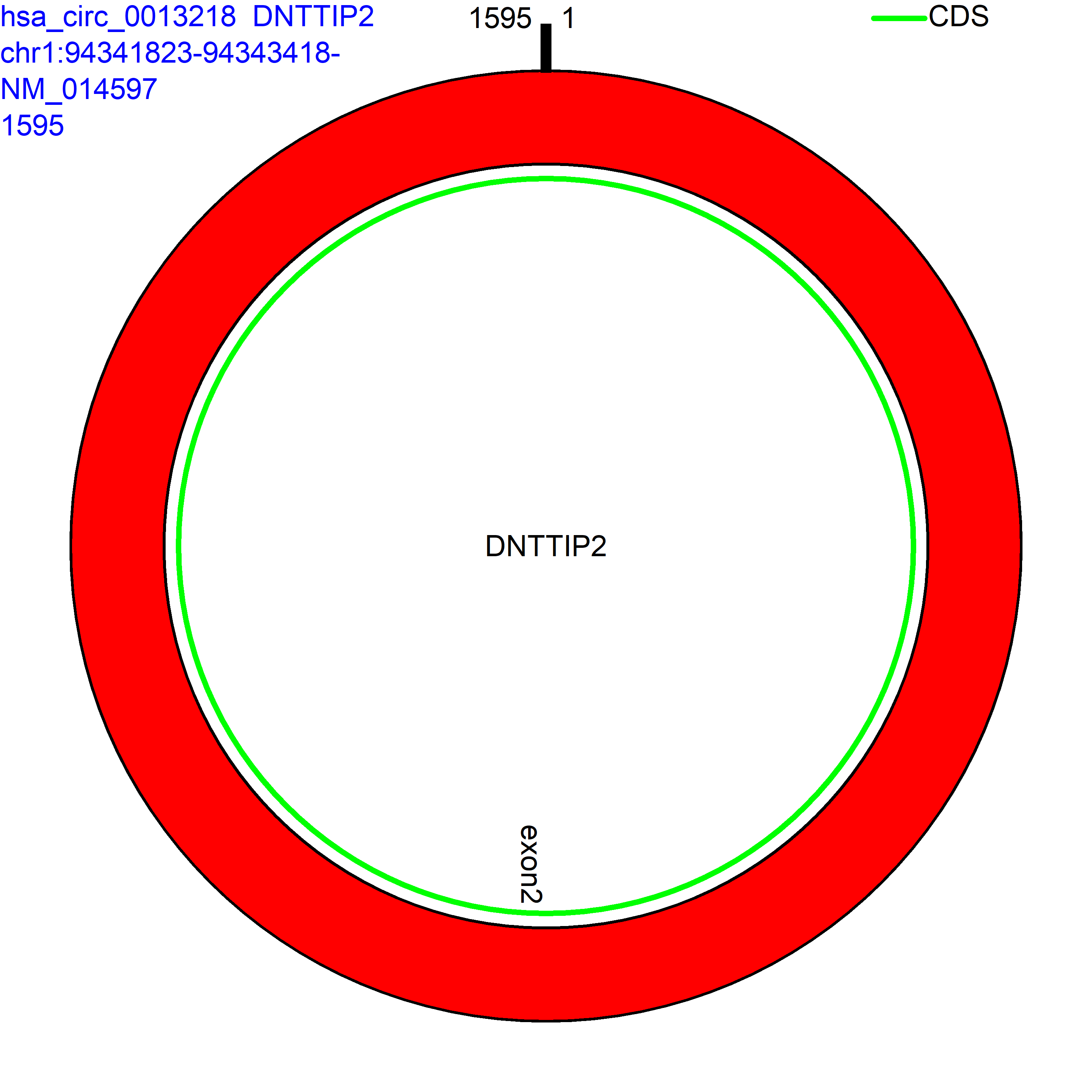

Supplement: Supplemental Information 4 [file peerj-11-16080-s004.zip › Figure2/circRNA.bmp]

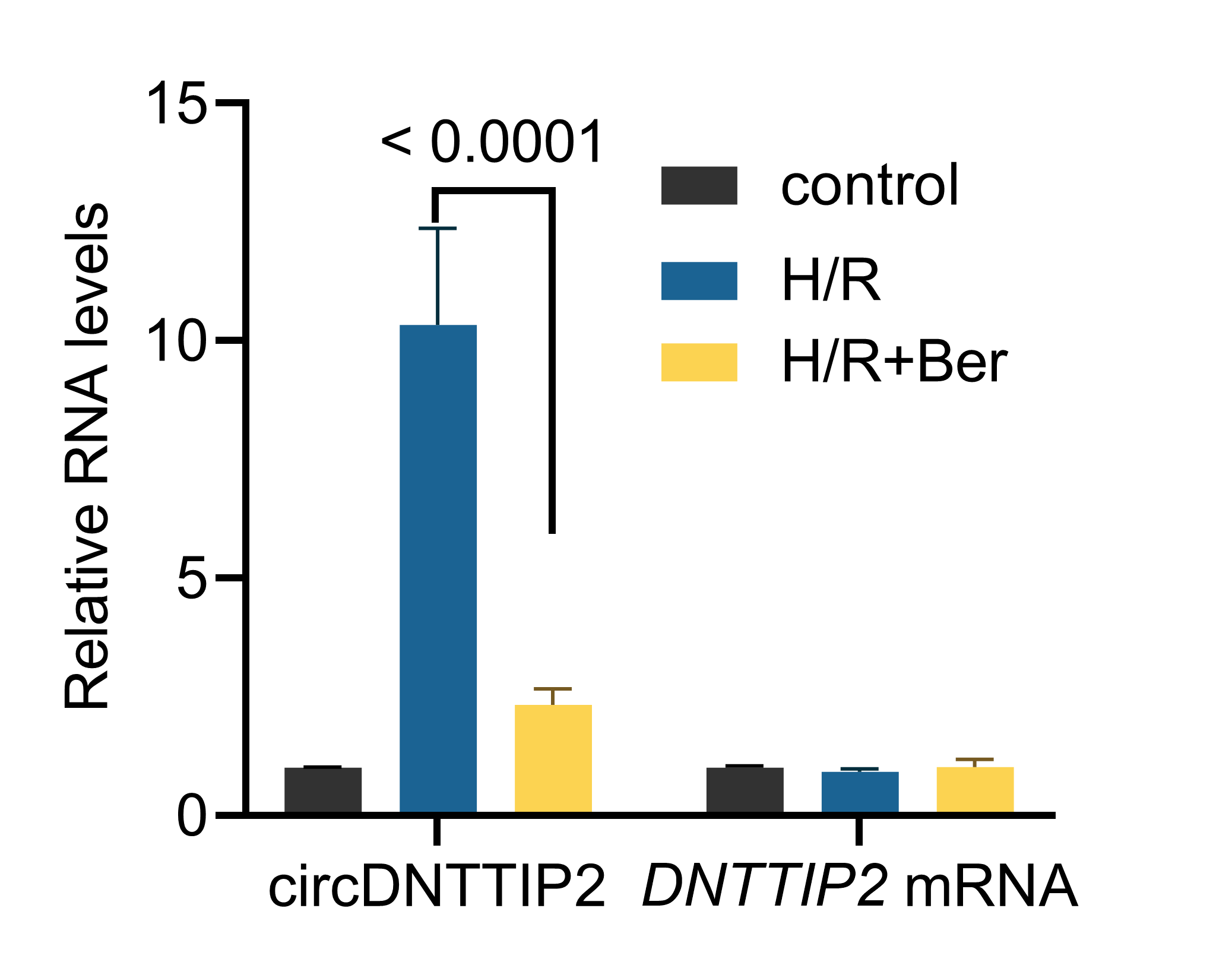

Supplement: Supplemental Information 4 [file peerj-11-16080-s004.zip › Figure2/Ber circRNA.tif]

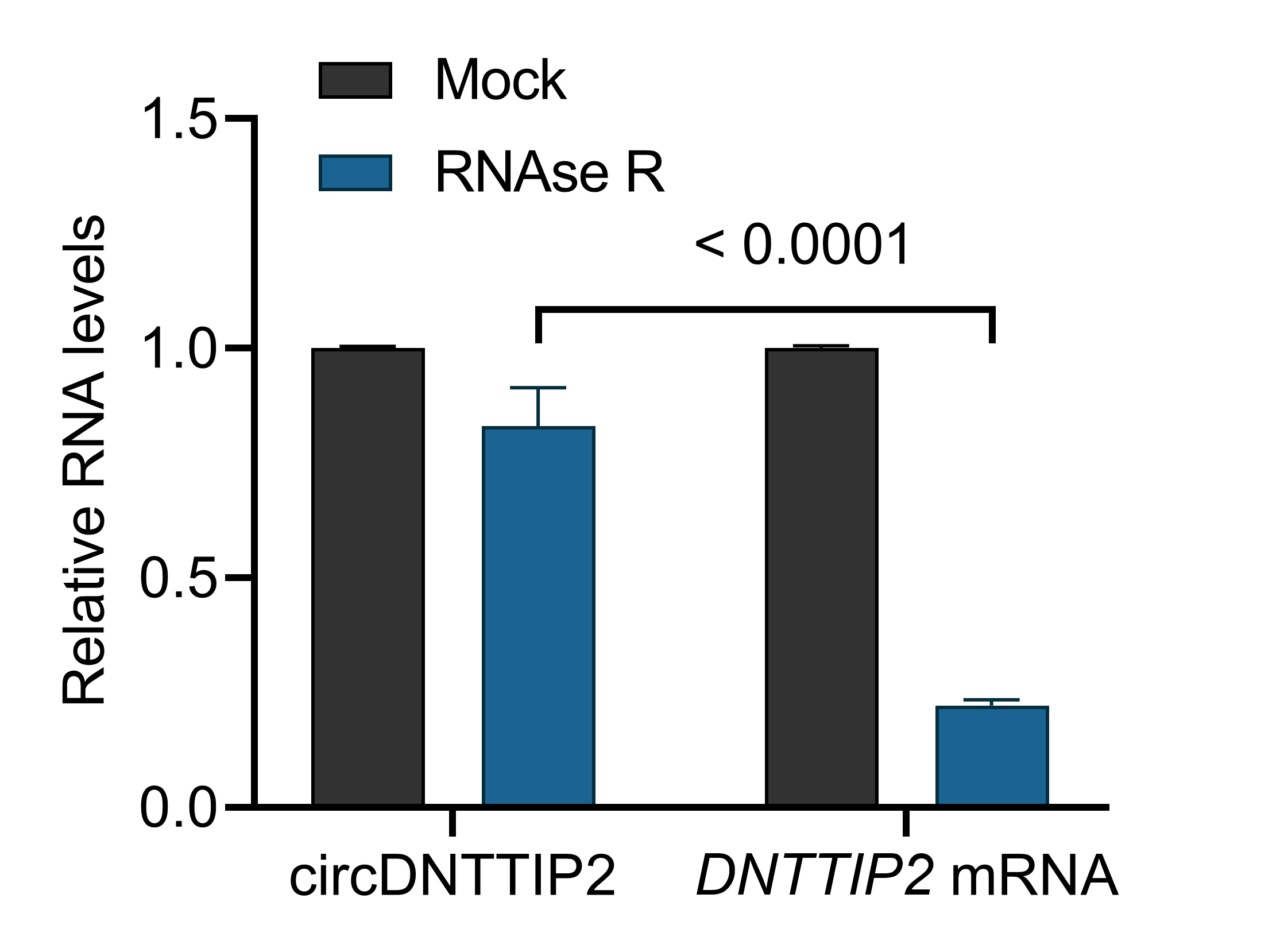

Supplement: Supplemental Information 4 [file peerj-11-16080-s004.zip › Figure2/RNase R.tif]

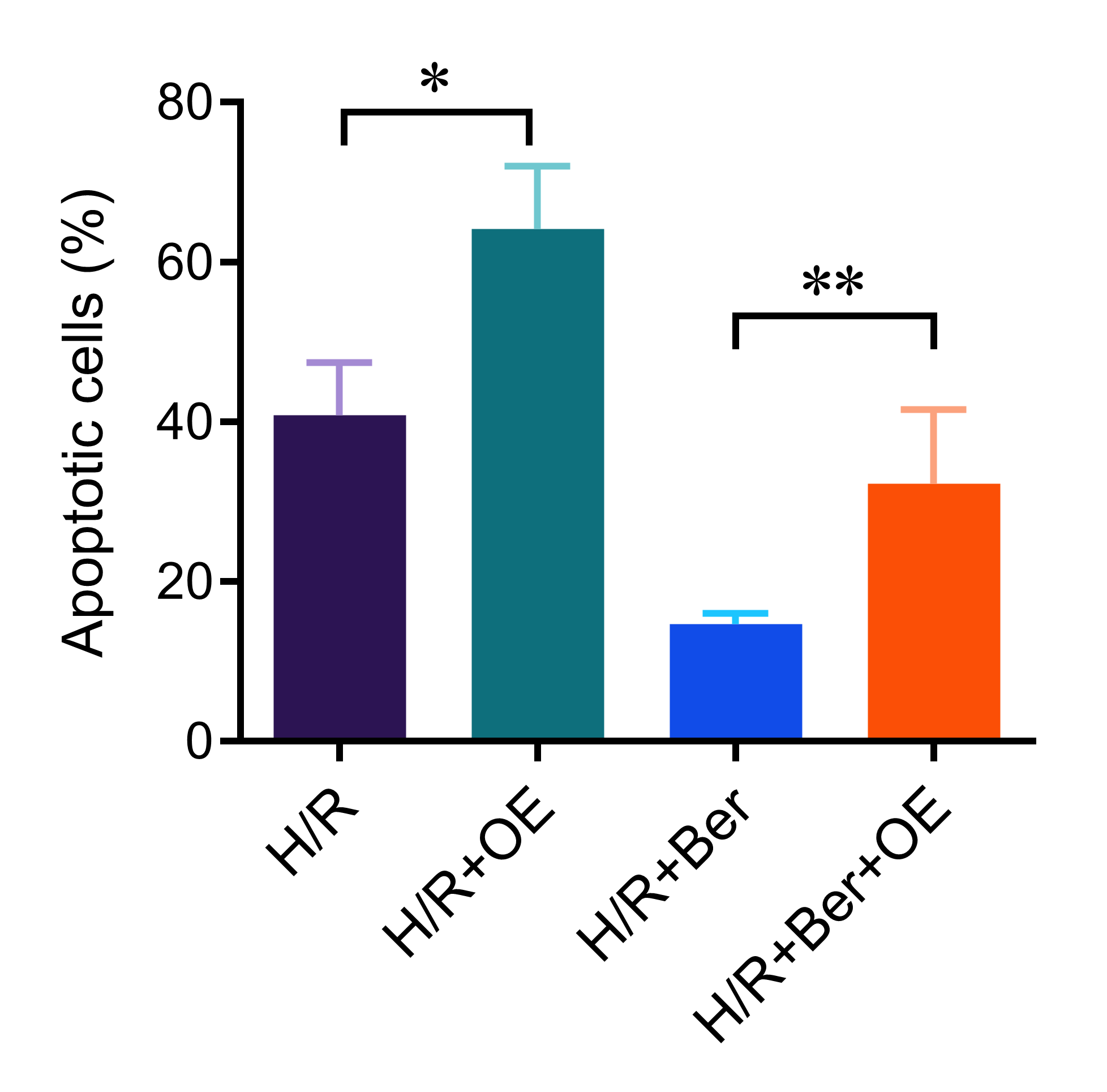

Supplement: Supplemental Information 5 [file peerj-11-16080-s005.zip › Figure3/apotosis 3.tif]

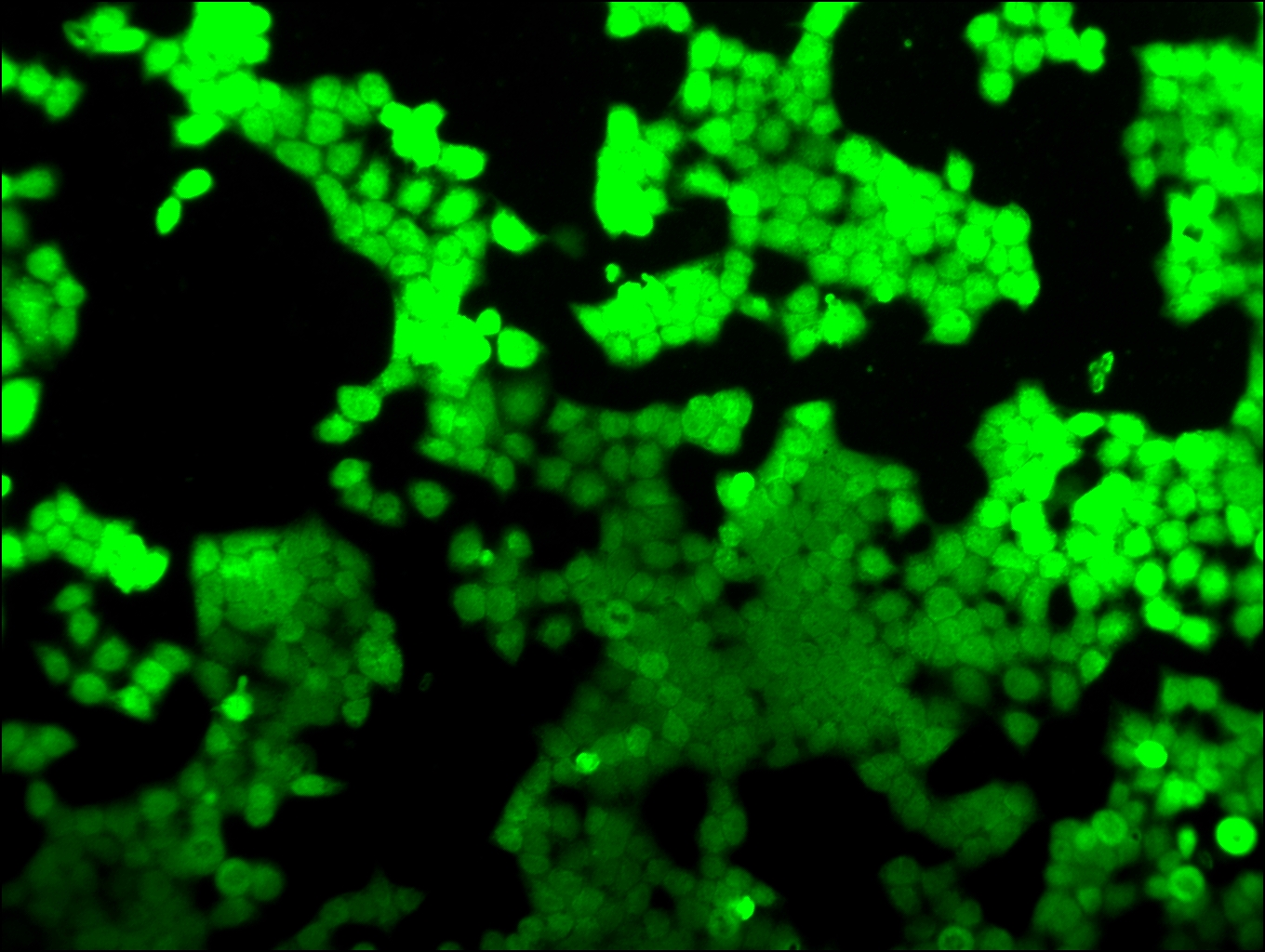

Supplement: Supplemental Information 5 [file peerj-11-16080-s005.zip › Figure3/HR OE Ber.jpg]

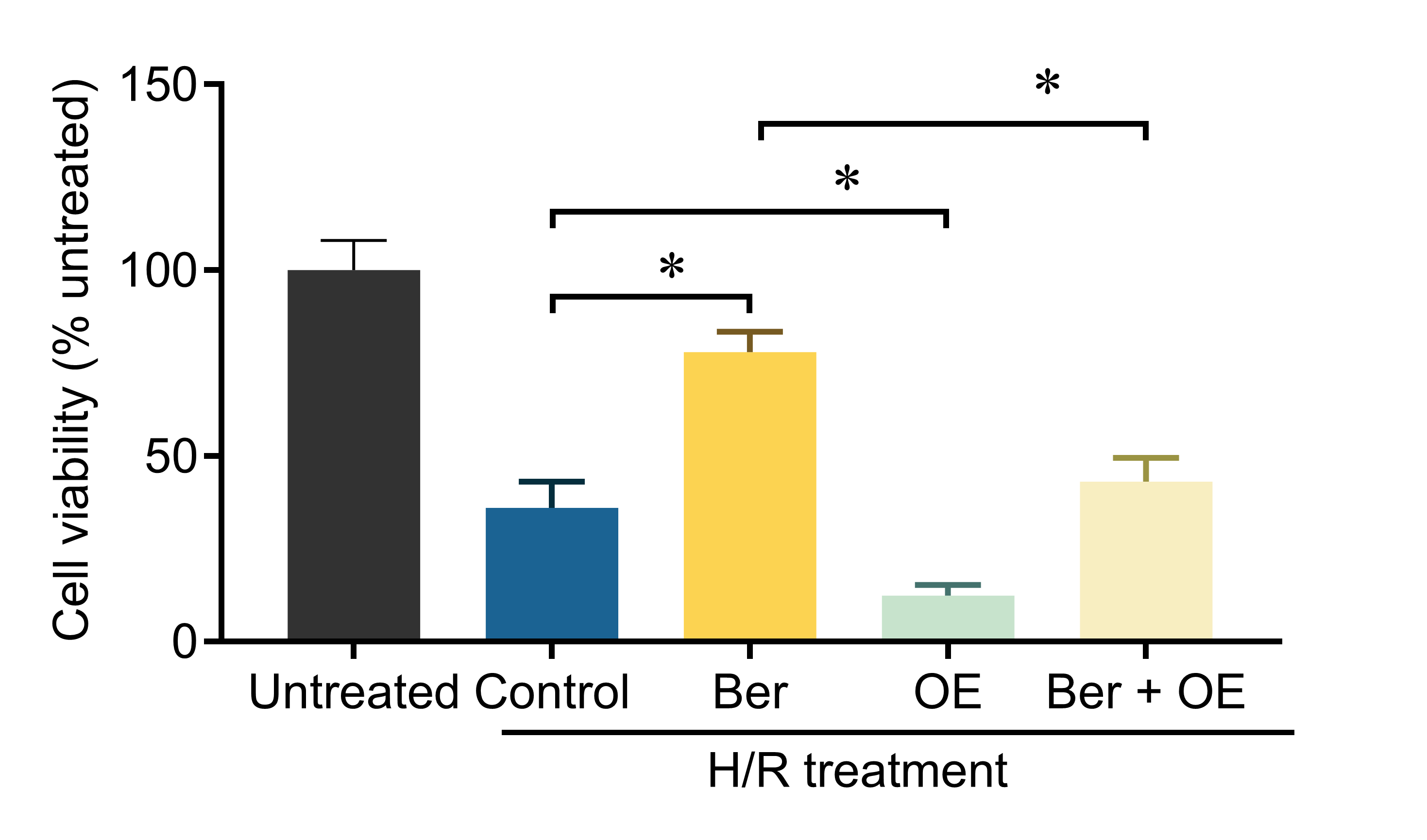

Supplement: Supplemental Information 5 [file peerj-11-16080-s005.zip › Figure3/cell viability ber OE.tif]

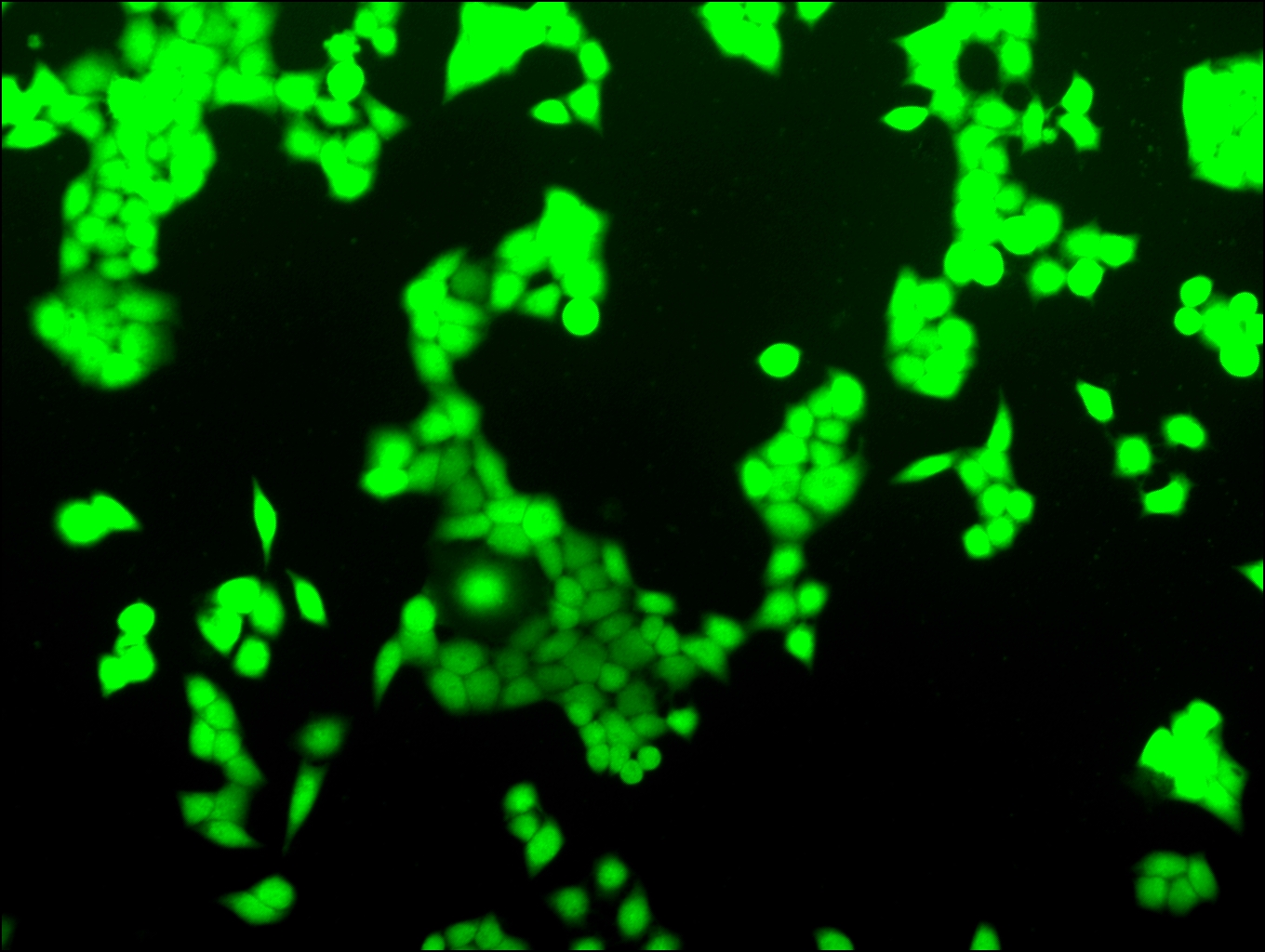

Supplement: Supplemental Information 5 [file peerj-11-16080-s005.zip › Figure3/HR.jpg]

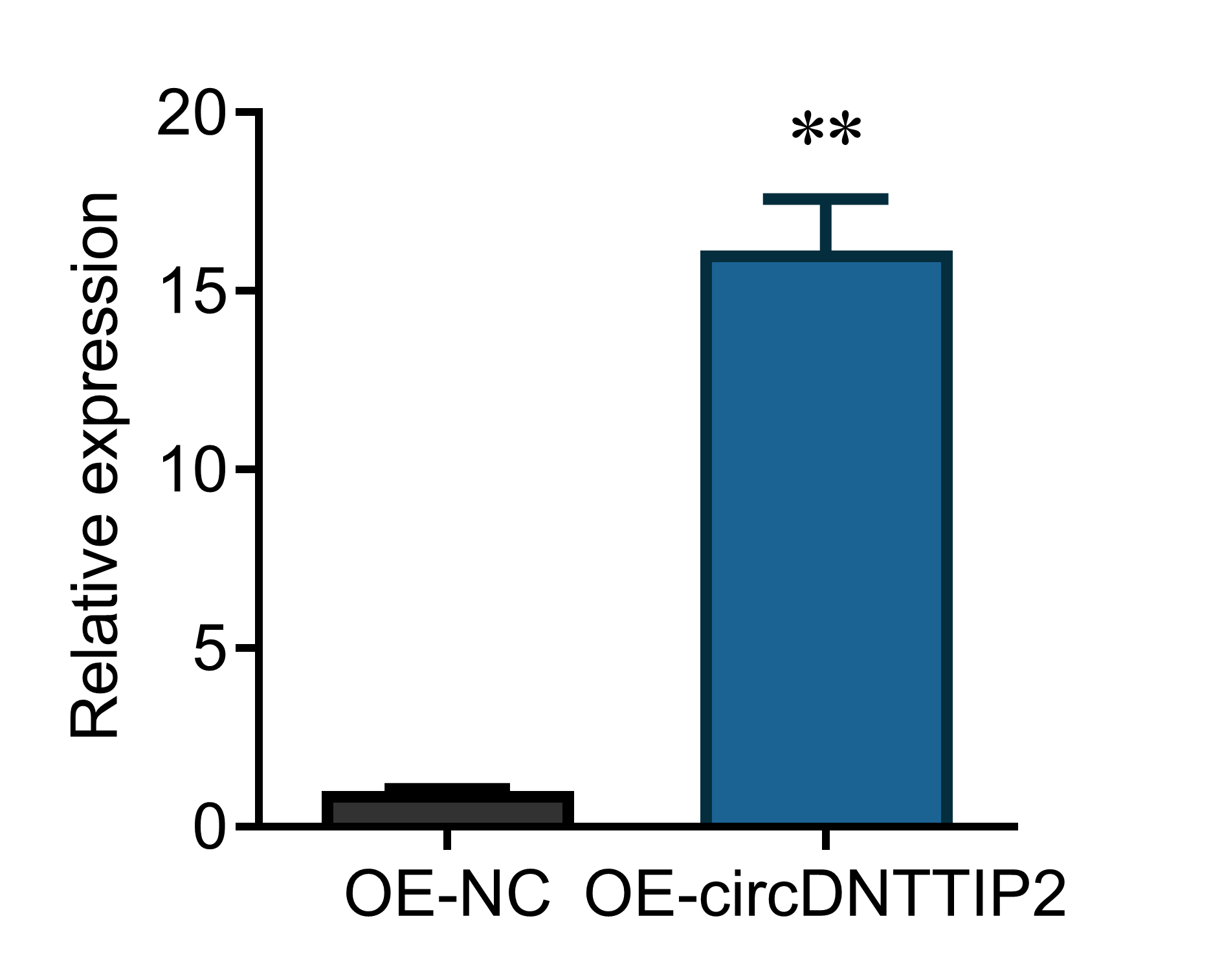

Supplement: Supplemental Information 5 [file peerj-11-16080-s005.zip › Figure3/OE.tif]

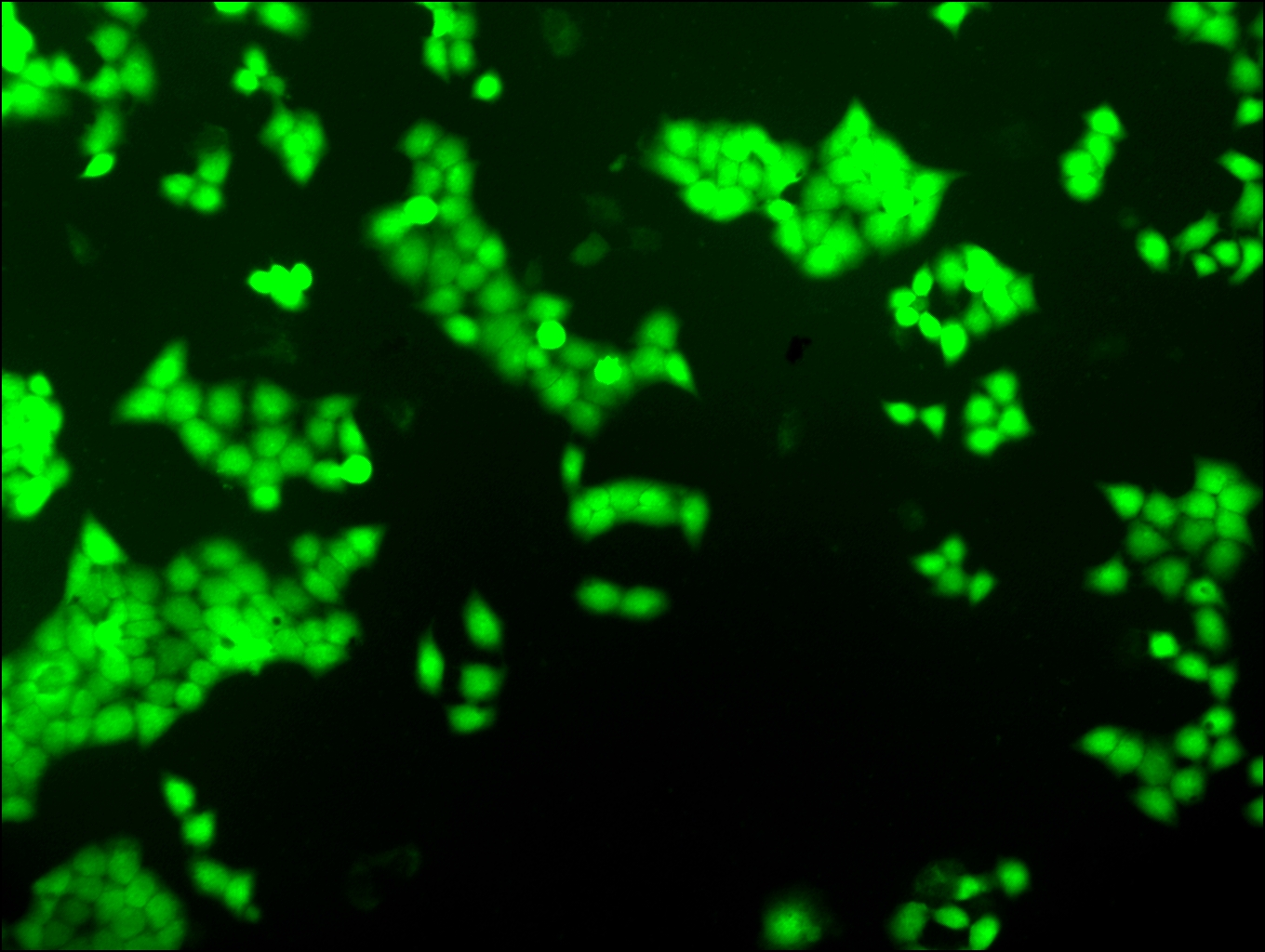

Supplement: Supplemental Information 5 [file peerj-11-16080-s005.zip › Figure3/HR + Ber.jpg]

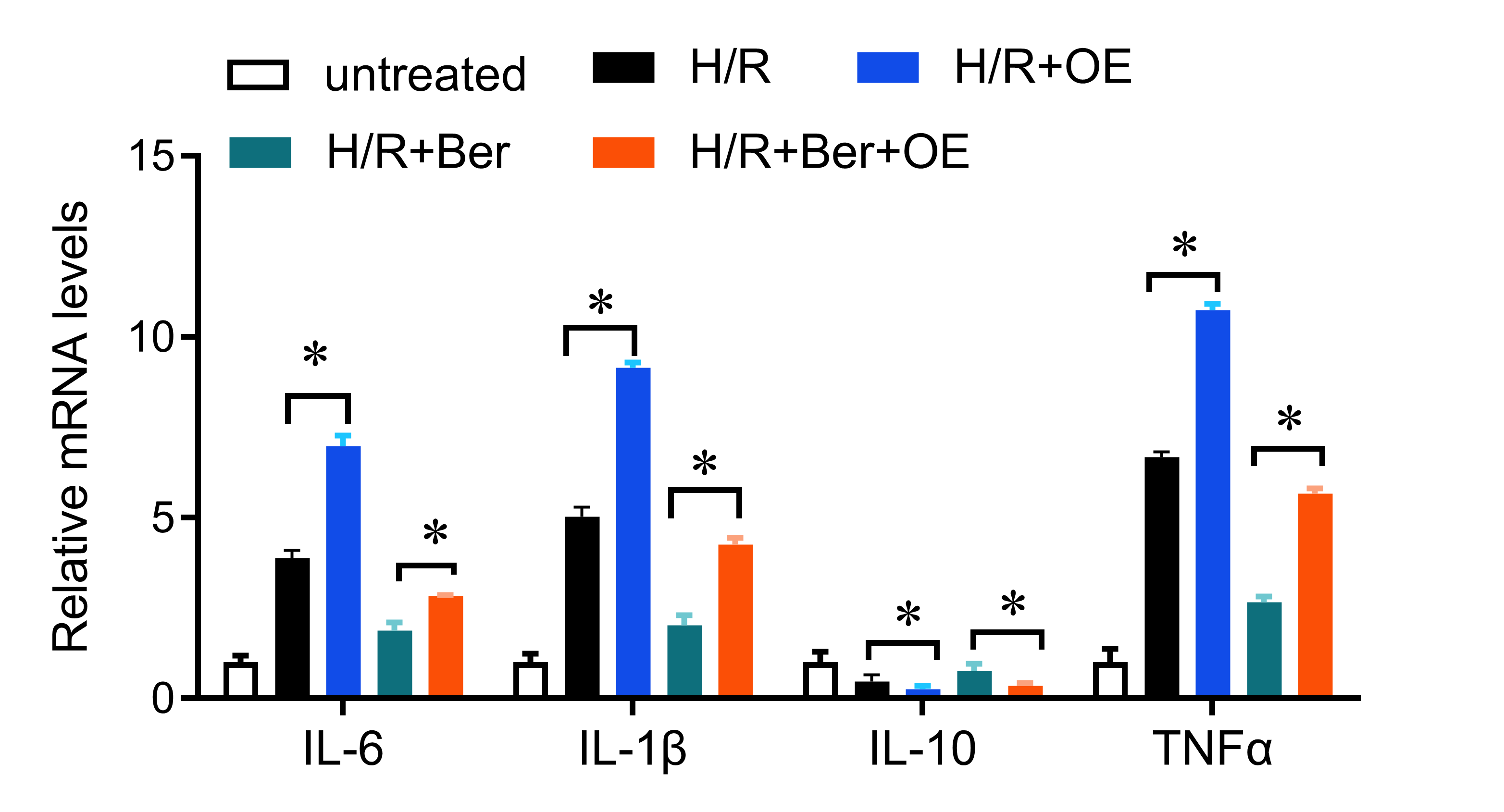

Supplement: Supplemental Information 5 [file peerj-11-16080-s005.zip › Figure3/mrna Figure3.tif]

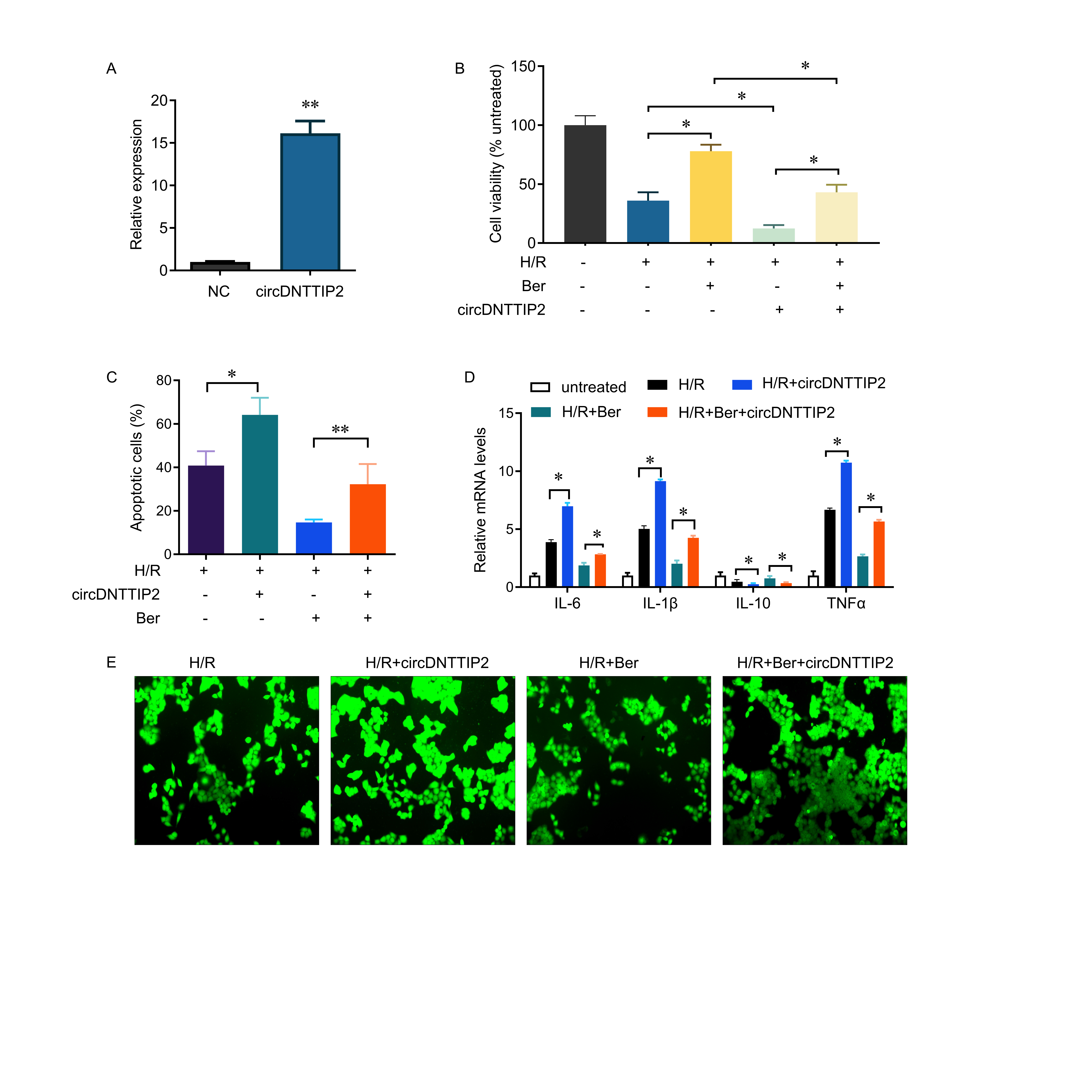

Supplement: Supplemental Information 5 [file peerj-11-16080-s005.zip › Figure3/Figure3-1.tif]

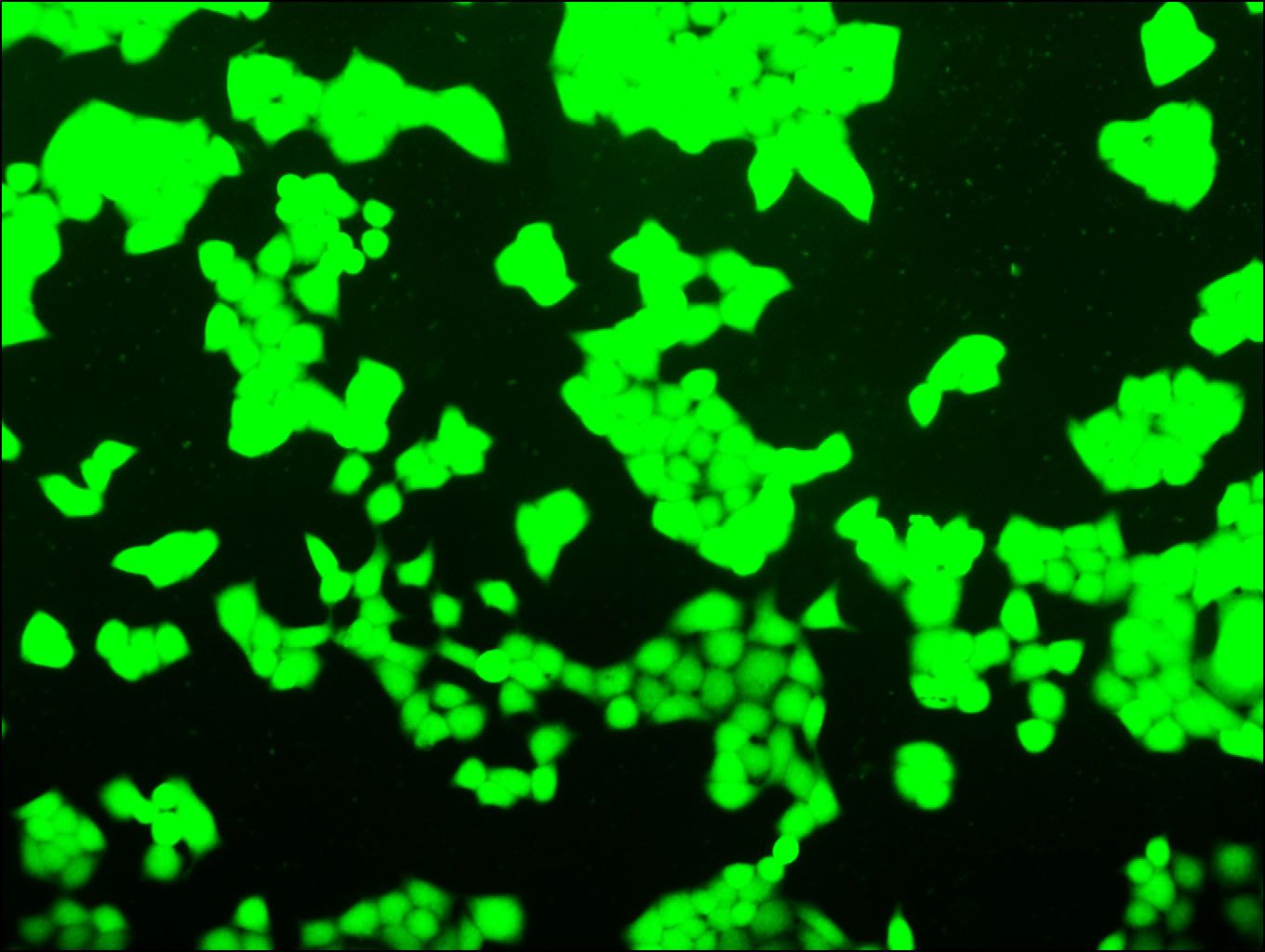

Supplement: Supplemental Information 5 [file peerj-11-16080-s005.zip › Figure3/HR OE.jpg]

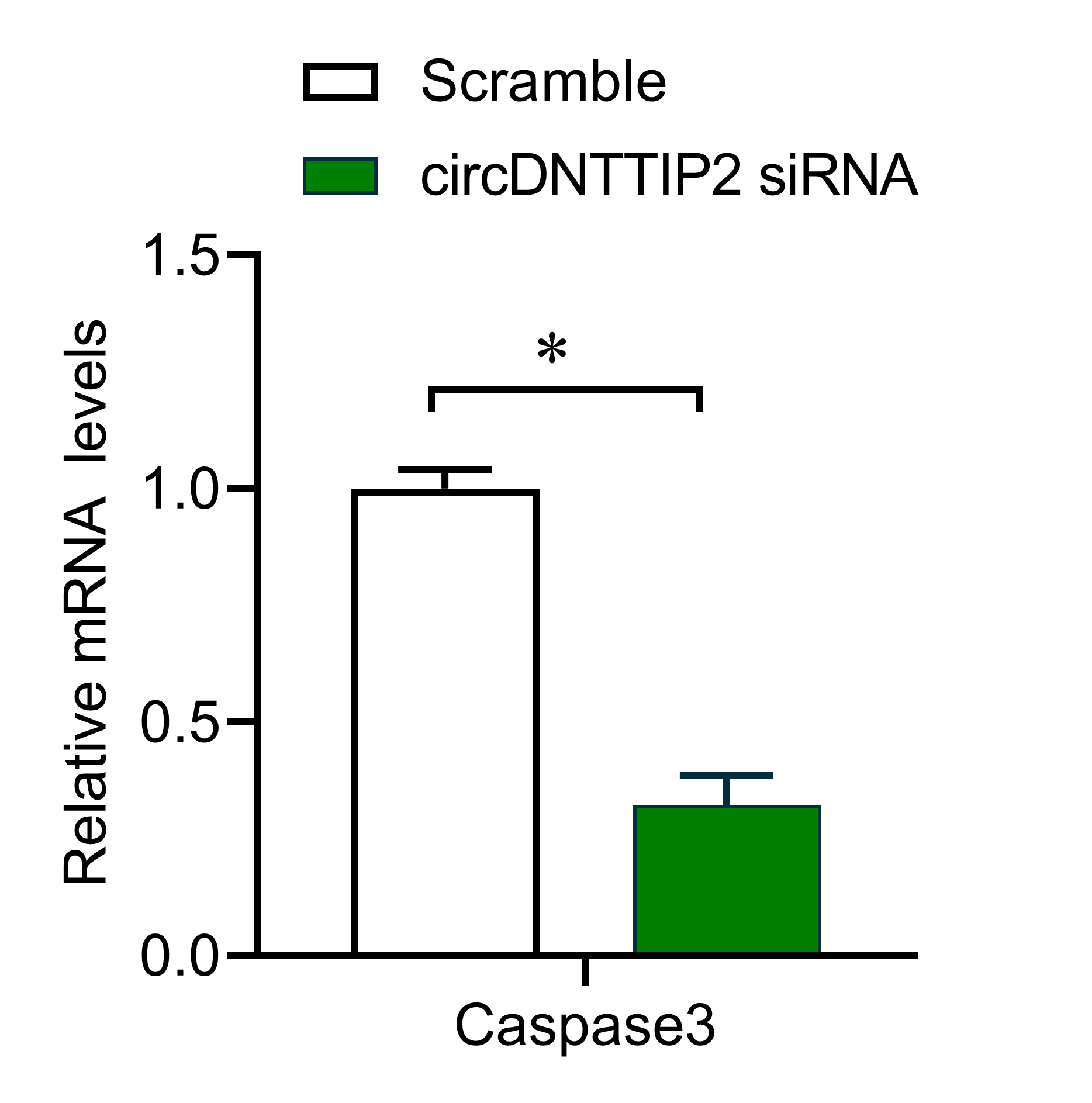

Supplement: Supplemental Information 6 [file peerj-11-16080-s006.zip › Figure4/circDNTTIP2 siRNA caspase 3.tif]

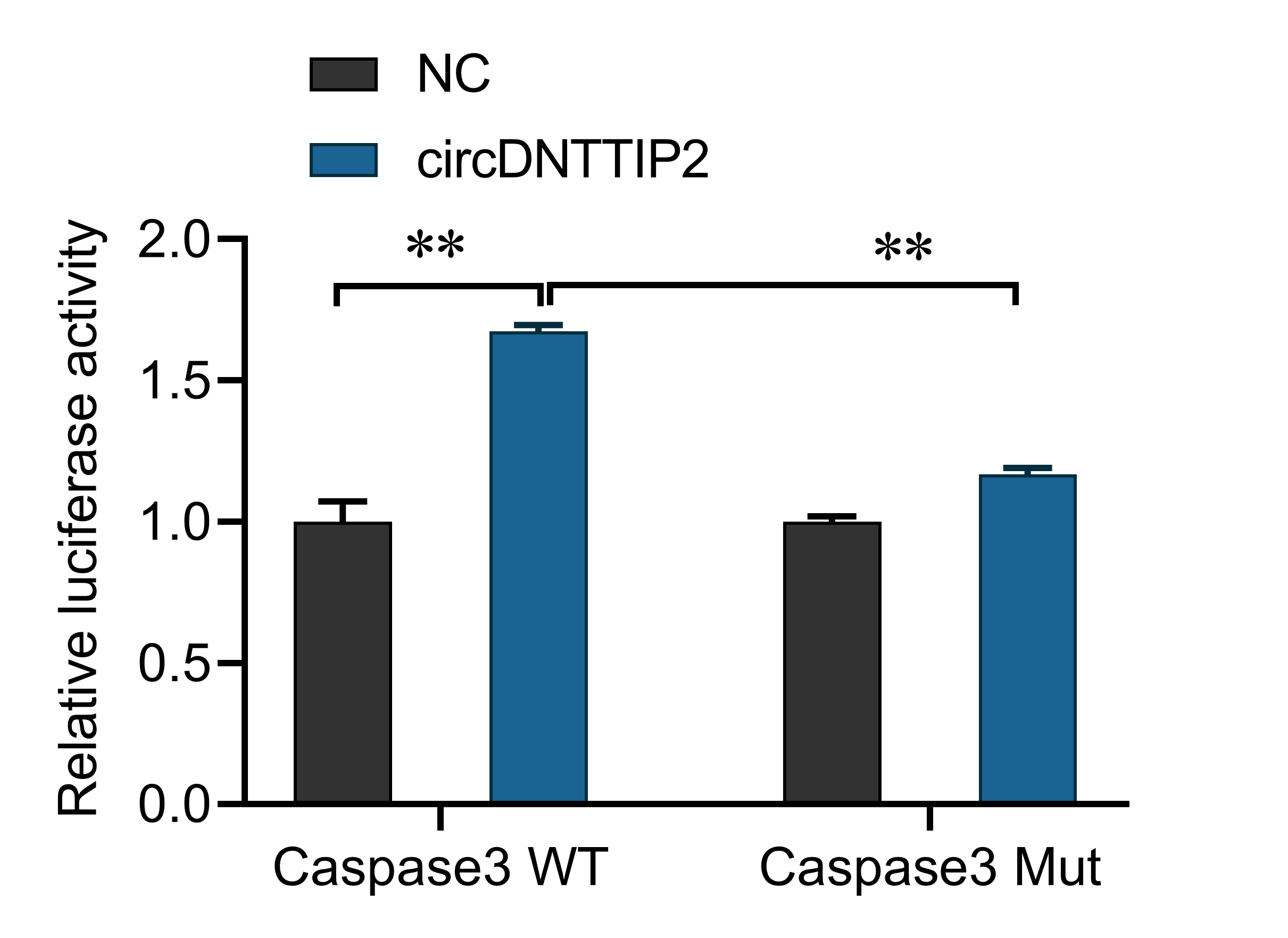

Supplement: Supplemental Information 6 [file peerj-11-16080-s006.zip › Figure4/Luciferase OE.tif]

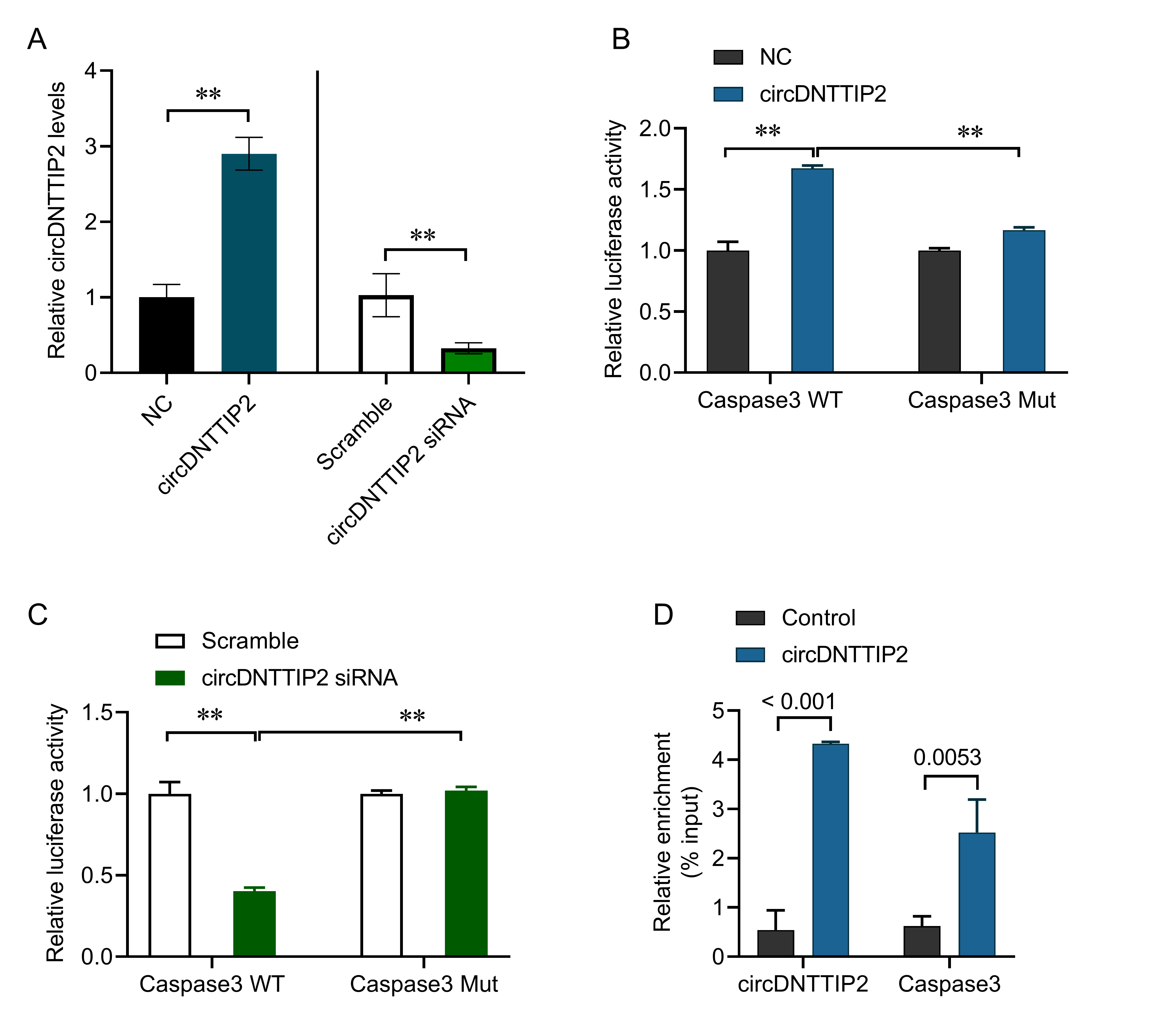

Supplement: Supplemental Information 6 [file peerj-11-16080-s006.zip › Figure4/Figure4.tif]

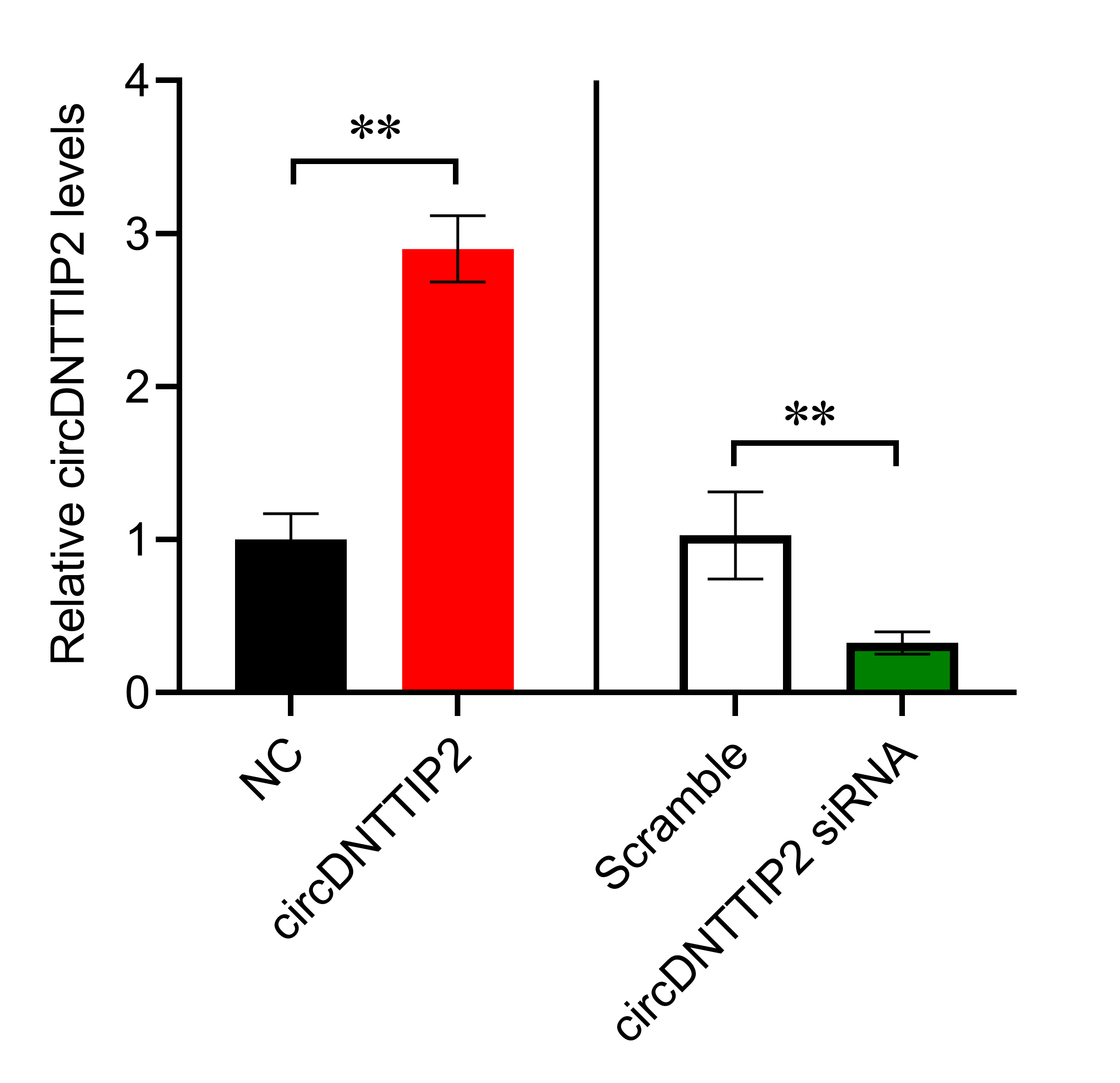

Supplement: Supplemental Information 6 [file peerj-11-16080-s006.zip › Figure4/Nested ANOVA.tif]

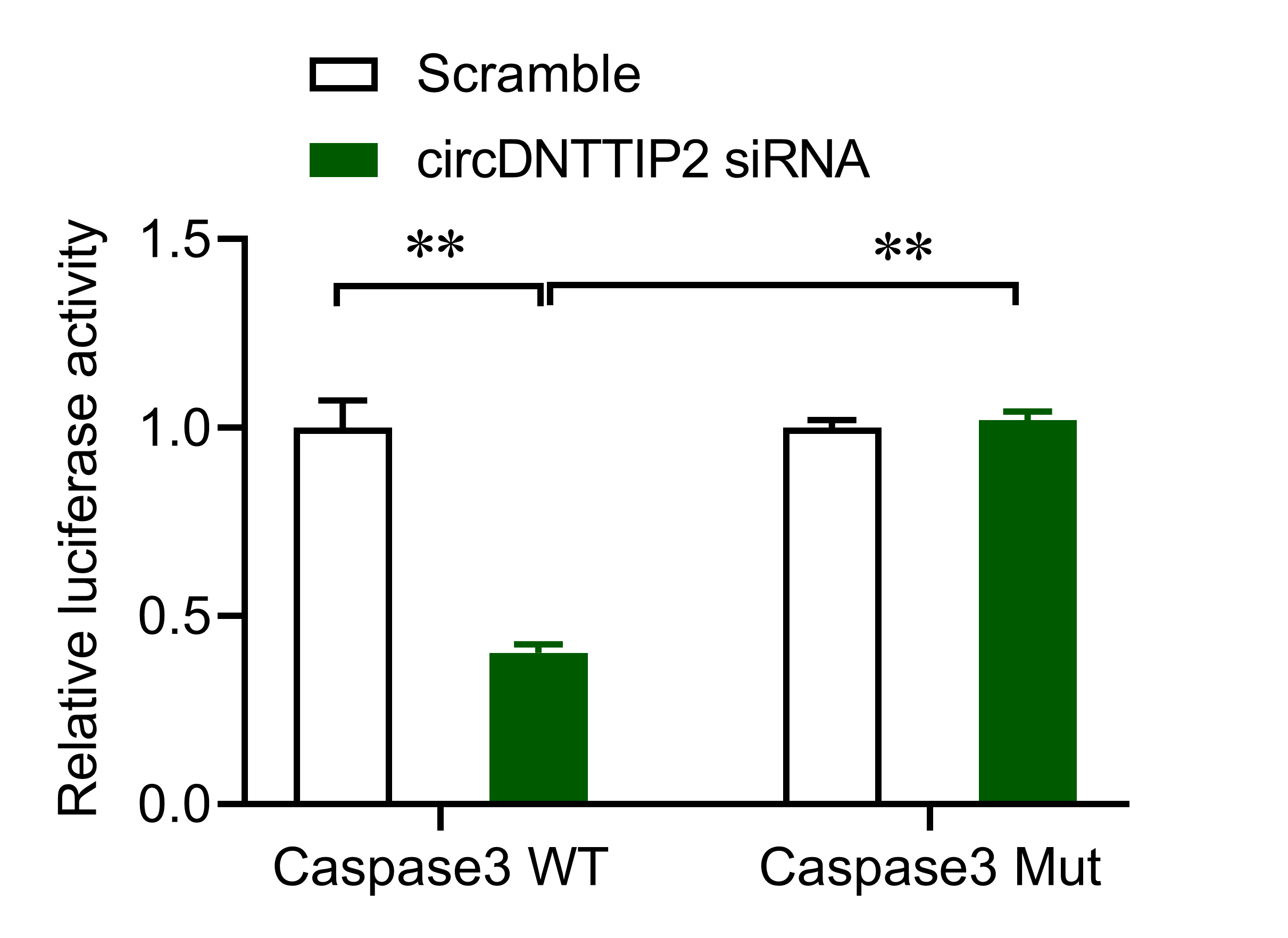

Supplement: Supplemental Information 6 [file peerj-11-16080-s006.zip › Figure4/Luciferase siRNA.tif]

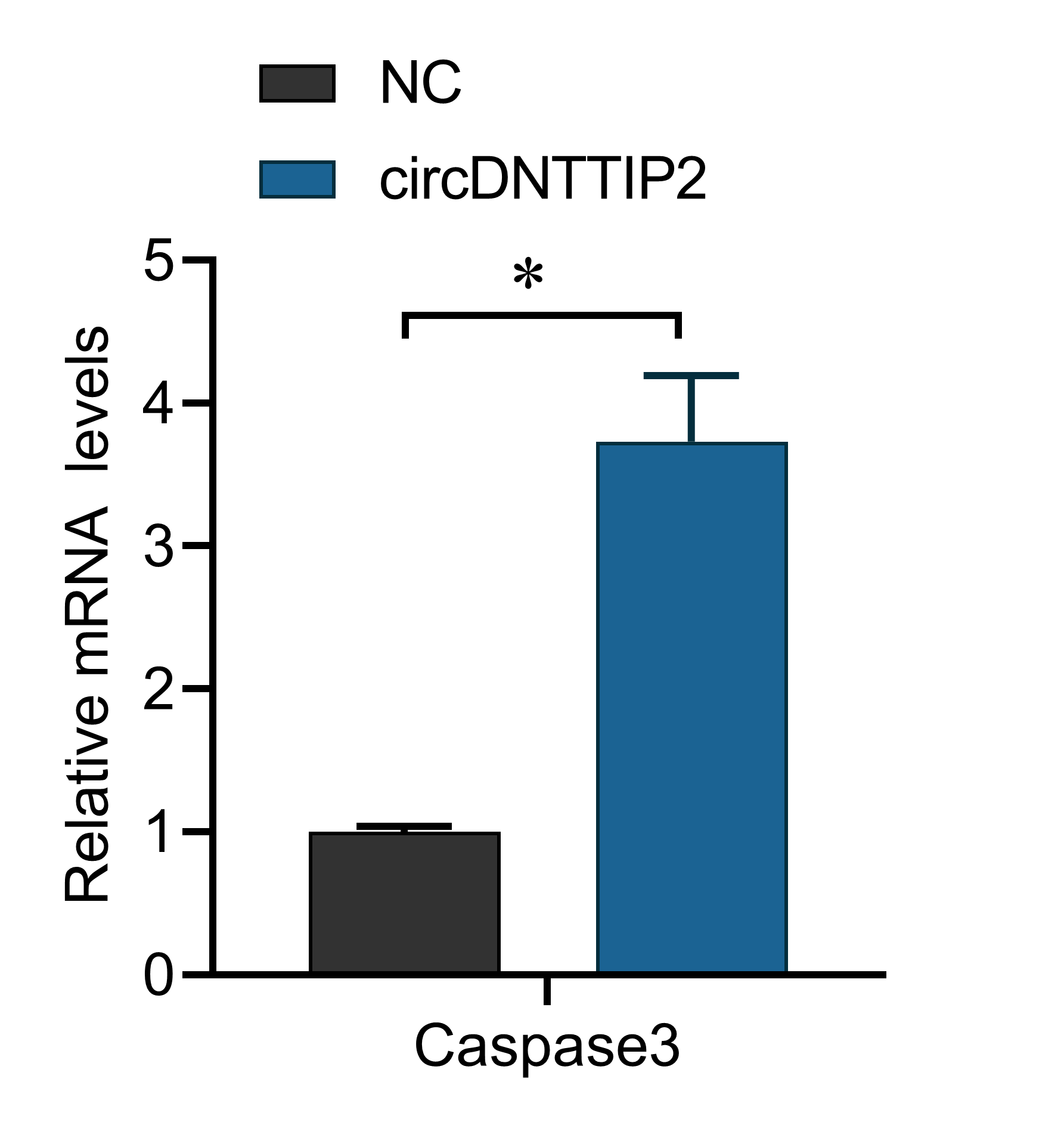

Supplement: Supplemental Information 6 [file peerj-11-16080-s006.zip › Figure4/circDNTTIP2 caspase 3.tif]

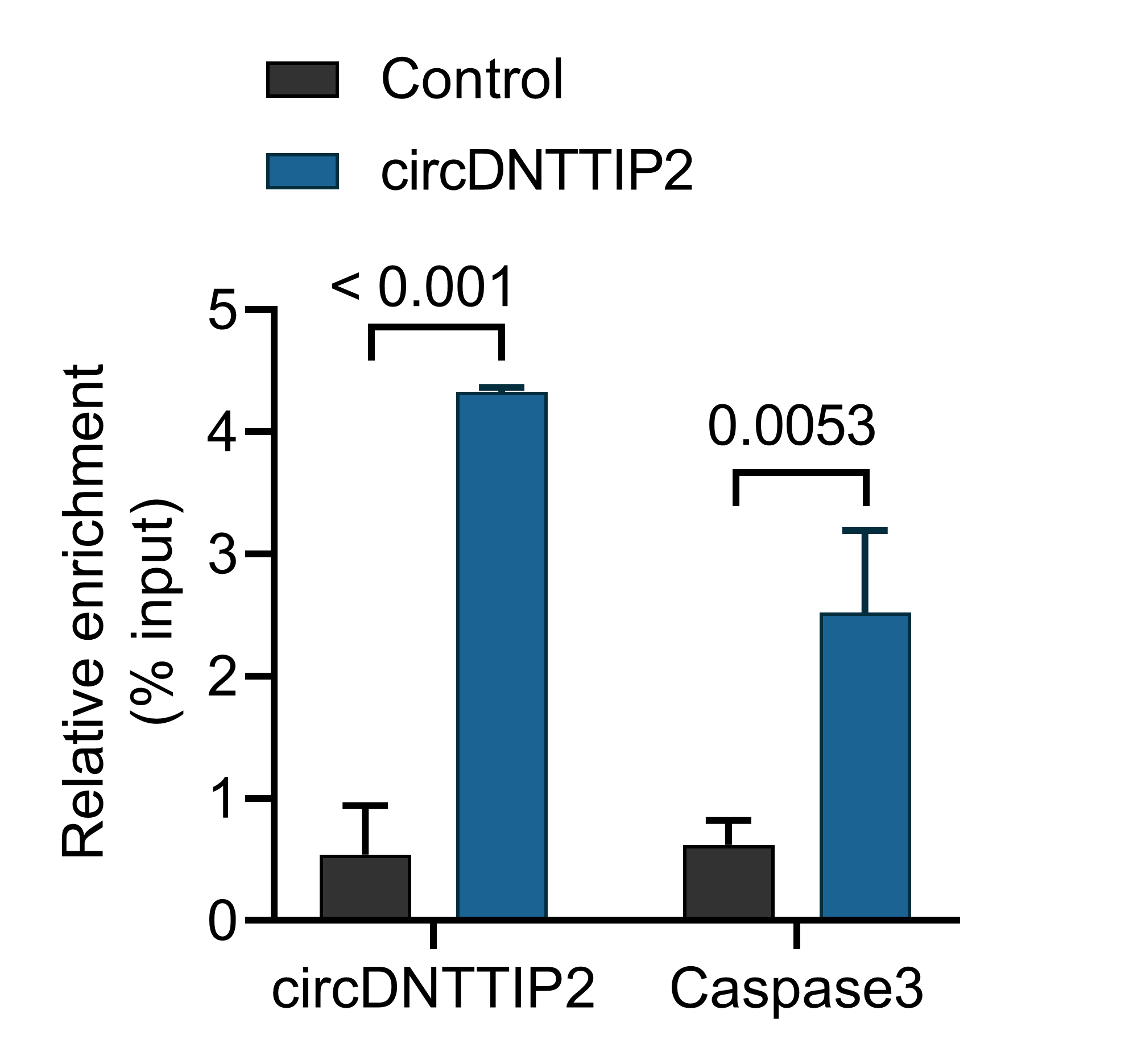

Supplement: Supplemental Information 6 [file peerj-11-16080-s006.zip › Figure4/caspase3 RIP.tif]

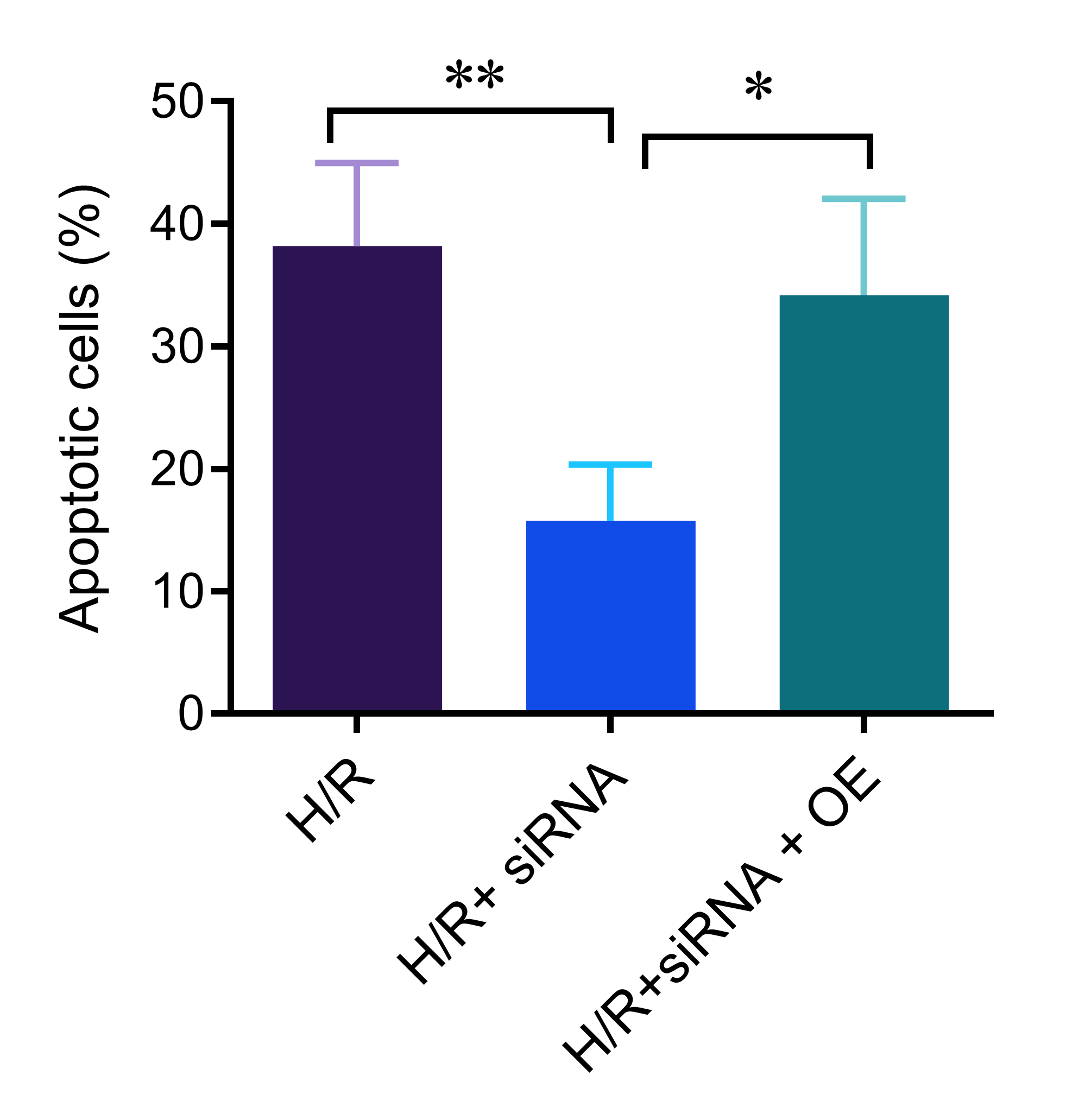

Supplement: Supplemental Information 7 [file peerj-11-16080-s007.zip › Figure5/apotosis 5.tif]

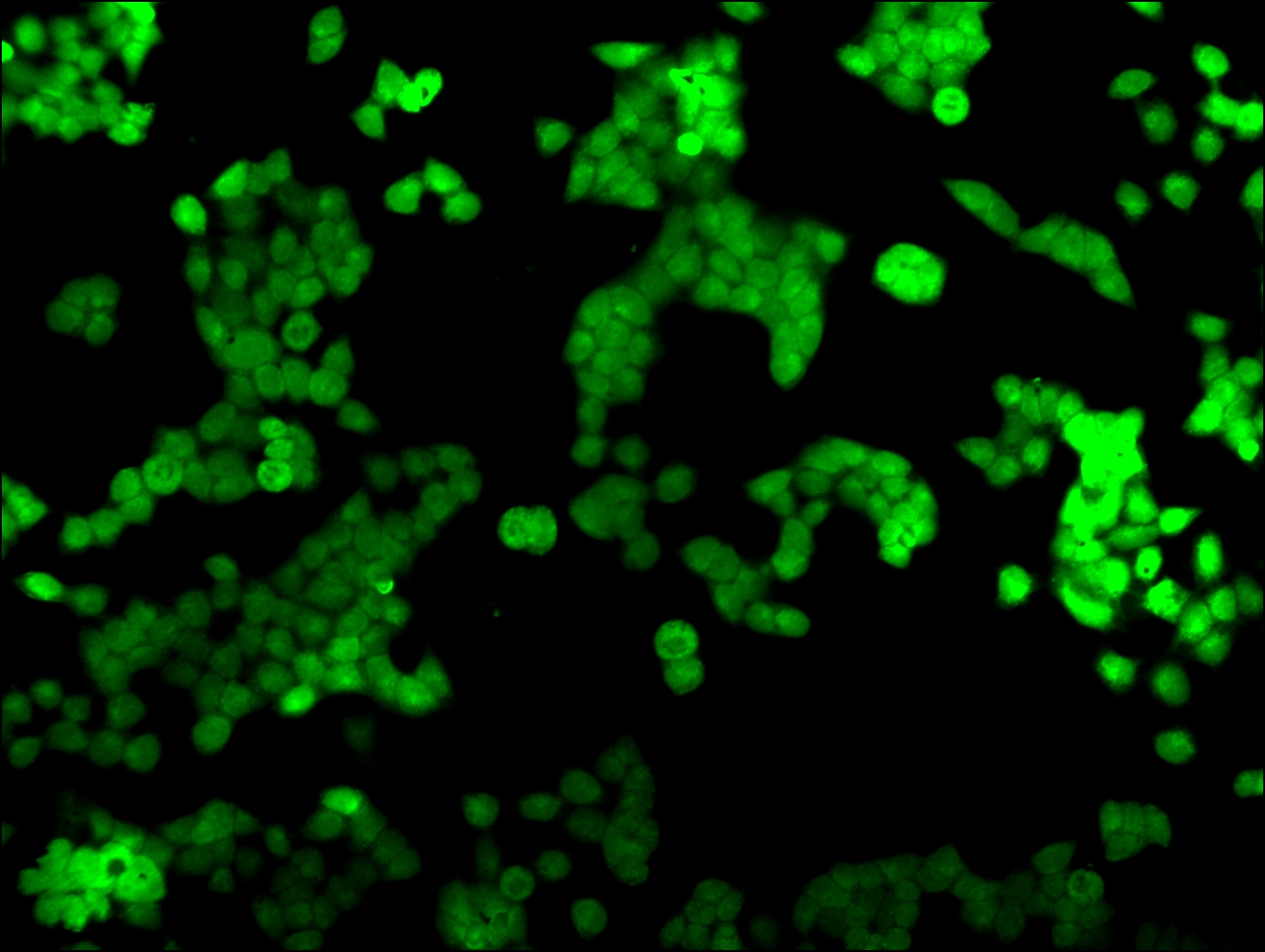

Supplement: Supplemental Information 7 [file peerj-11-16080-s007.zip › Figure5/siRNA.jpg]

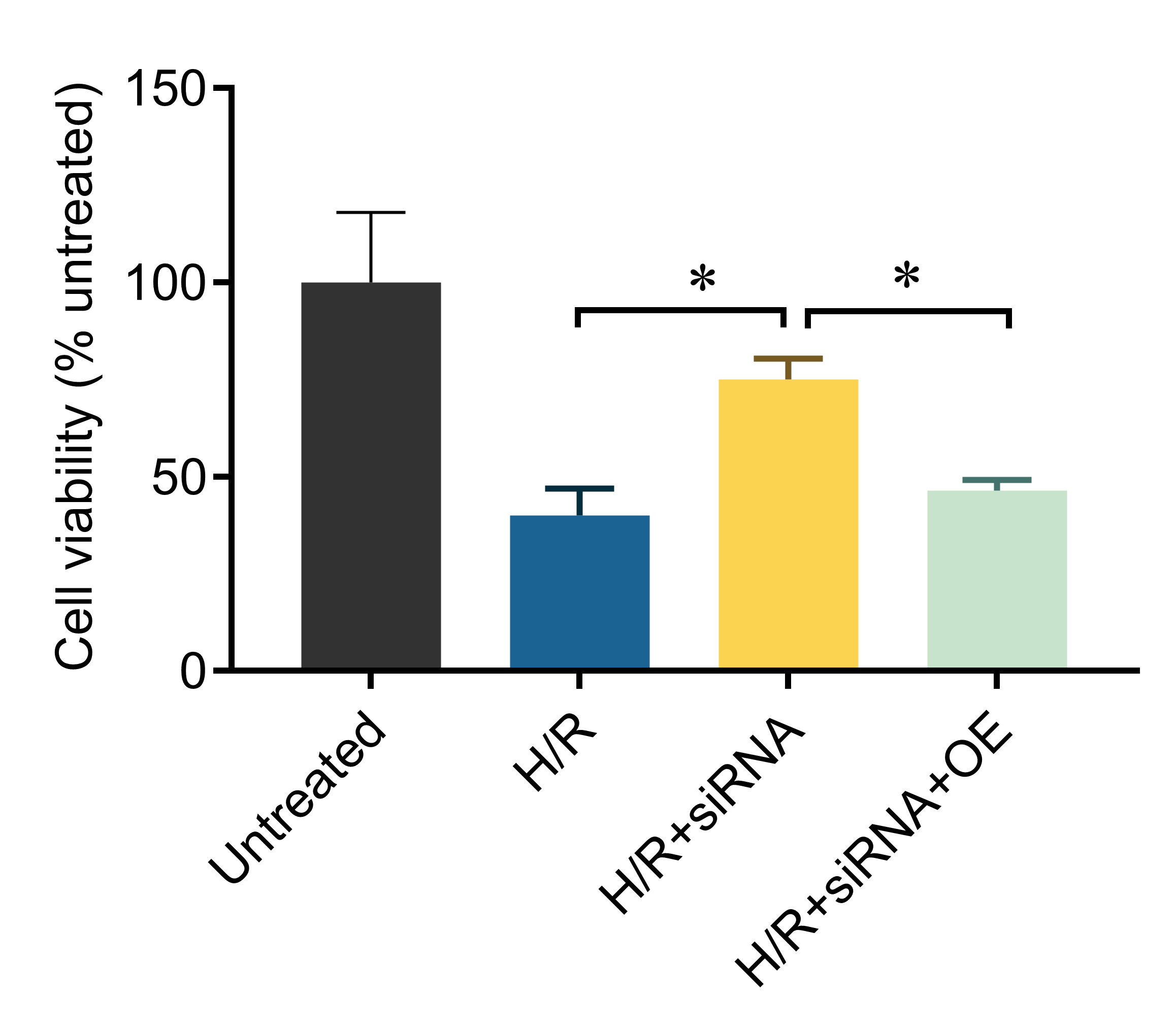

Supplement: Supplemental Information 7 [file peerj-11-16080-s007.zip › Figure5/cell viability siRNA OE.tif]

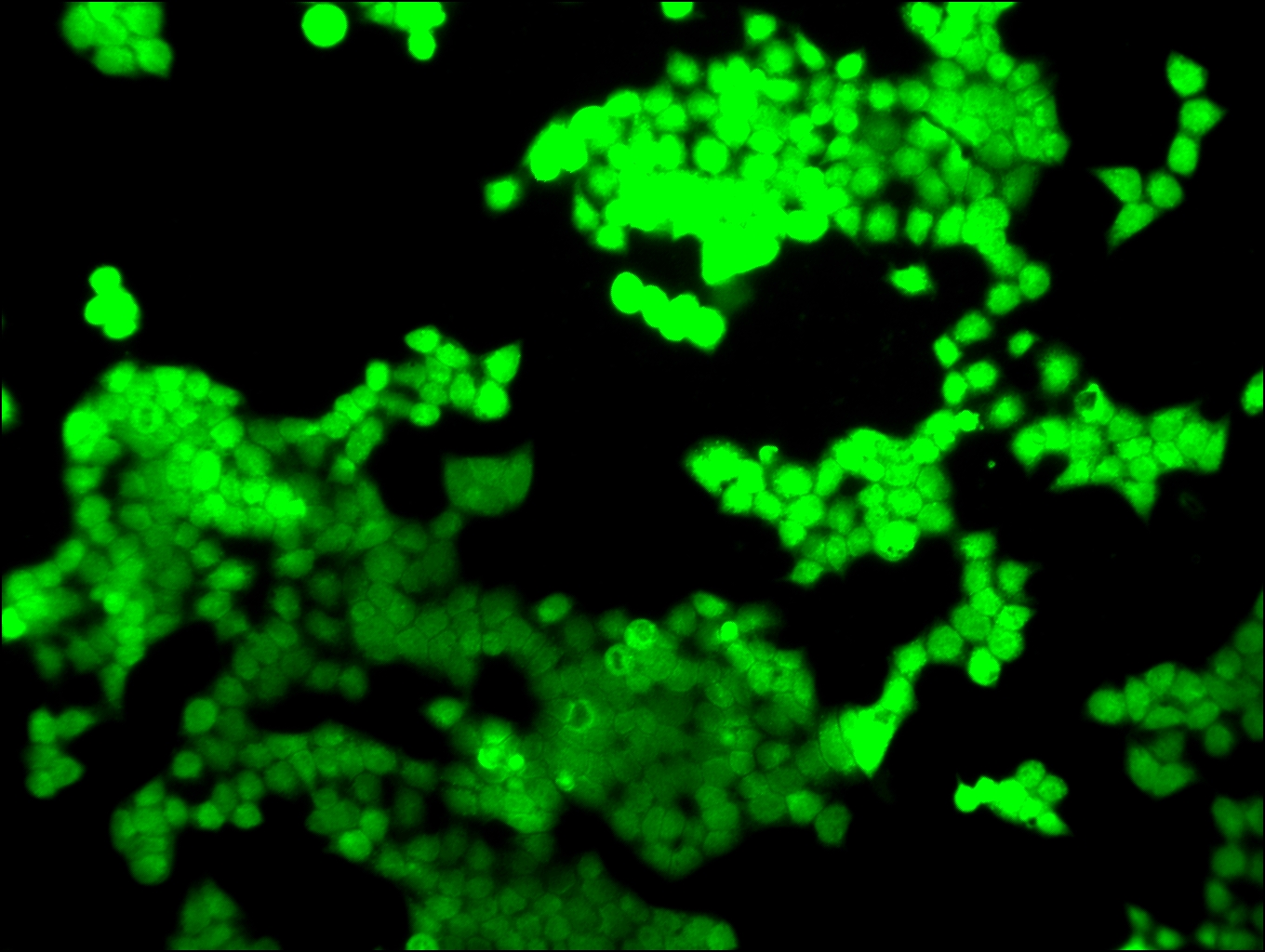

Supplement: Supplemental Information 7 [file peerj-11-16080-s007.zip › Figure5/siRNA OE.jpg]

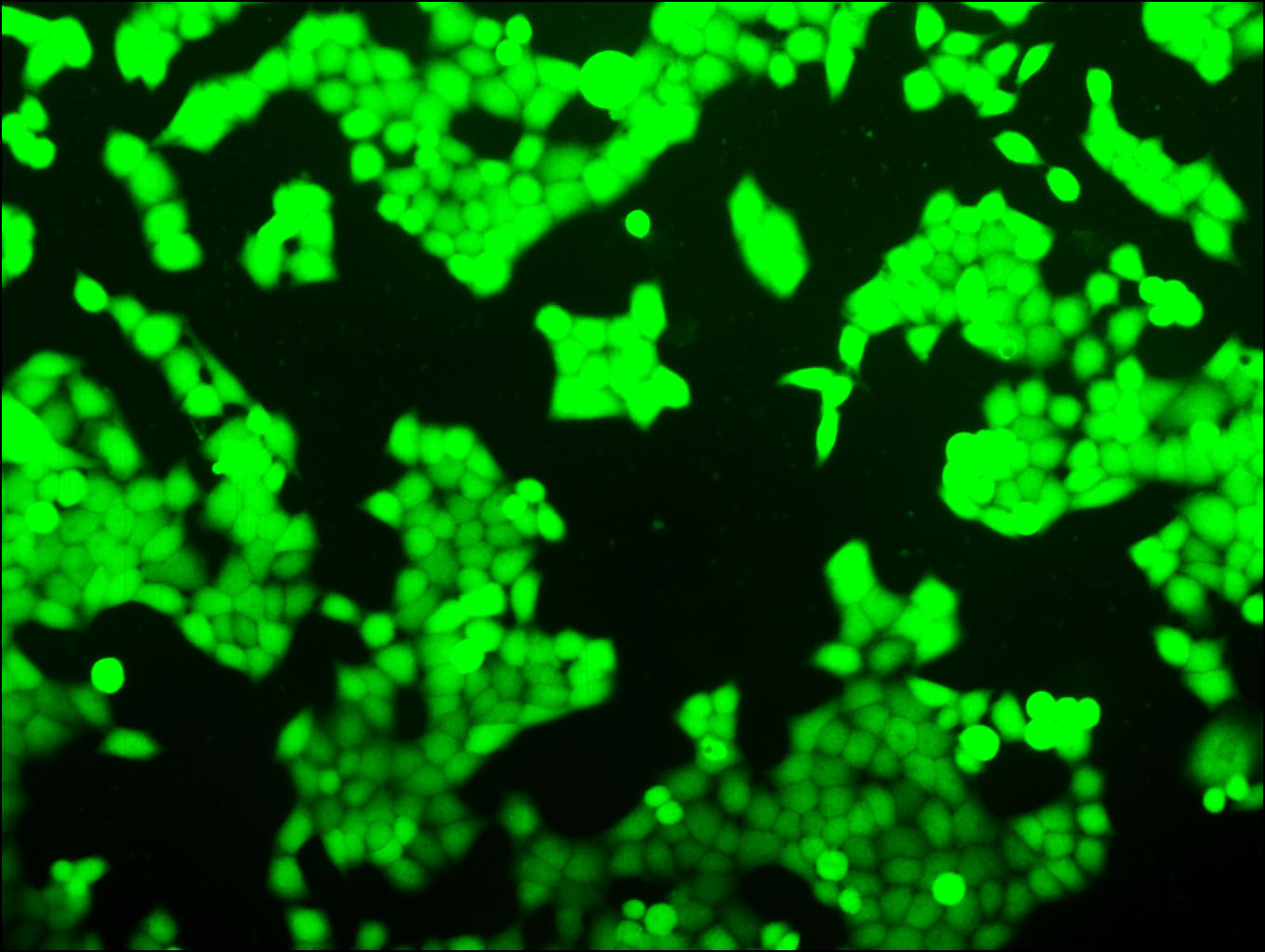

Supplement: Supplemental Information 7 [file peerj-11-16080-s007.zip › Figure5/HR.jpg]

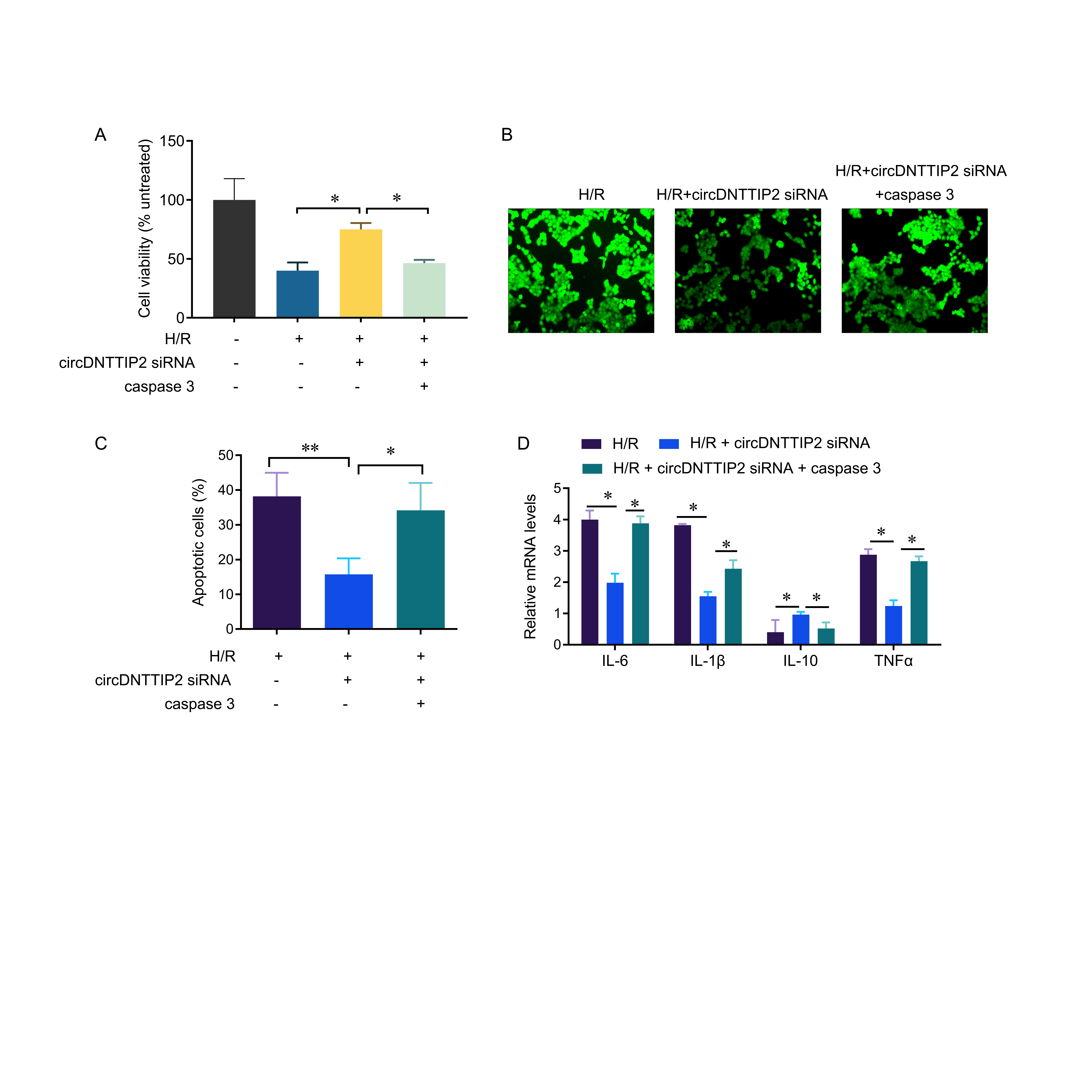

Supplement: Supplemental Information 7 [file peerj-11-16080-s007.zip › Figure5/Figure5-1.tif]

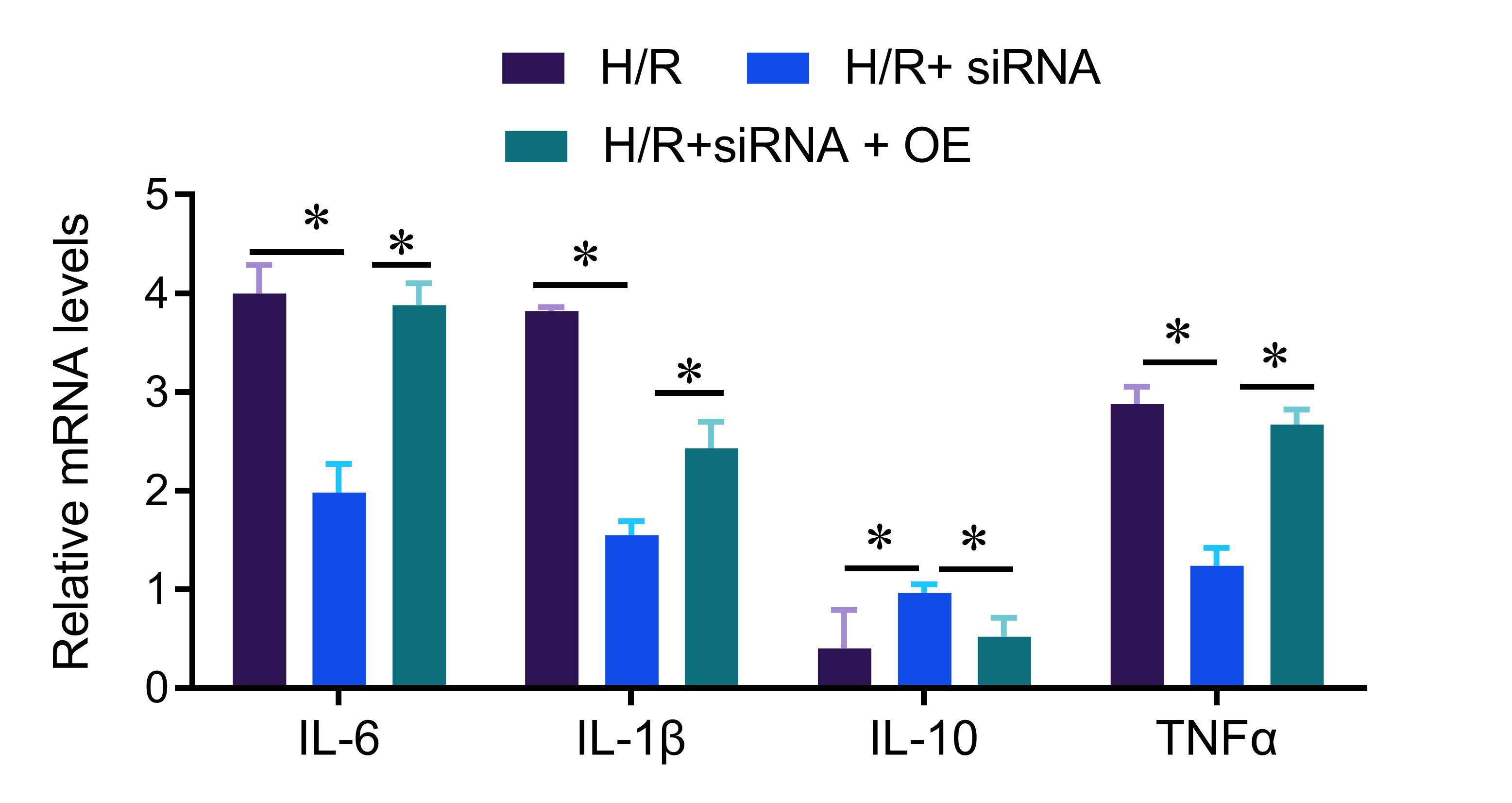

Supplement: Supplemental Information 7 [file peerj-11-16080-s007.zip › Figure5/mrna Figure5.tif]
